# Monoallelic Loss of the Imprinted Gene *Grb10* Promotes Tumor Formation in Irradiated *Nf1^+/-^* Mice

**DOI:** 10.1371/journal.pgen.1005235

**Published:** 2015-05-22

**Authors:** Rana Mroue, Brian Huang, Steve Braunstein, Ari J. Firestone, Jean L. Nakamura

**Affiliations:** 1 Department of Radiation Oncology, University of California, San Francisco, San Francisco, California, United States of America; 2 Department of Pediatrics, University of California, San Francisco, San Francisco, California, United States of America; UNITED STATES

## Abstract

Imprinted genes are expressed from only one parental allele and heterozygous loss involving the expressed allele is sufficient to produce complete loss of protein expression. Genetic alterations are common in tumorigenesis but the role of imprinted genes in this process is not well understood. In earlier work we mutagenized mice heterozygous for the Neurofibromatosis I tumor suppressor gene (*NF1*) to model radiotherapy-associated second malignant neoplasms that arise in irradiated NF1 patients. Expression analysis of tumor cell lines established from our mouse models identified *Grb10* expression as widely absent. *Grb10* is an imprinted gene and polymorphism analysis of cell lines and primary tumors demonstrates that the expressed allele is commonly lost in diverse *Nf1* mutant tumors arising in our mouse models. We performed functional studies to test whether *Grb10* restoration or loss alter fundamental features of the tumor growth. Restoring *Grb10* in *Nf1* mutant tumors decreases proliferation, decreases soft agar colony formation and downregulates Ras signaling. Conversely, *Grb10* silencing in untransformed mouse embryo fibroblasts significantly increased cell proliferation and increased Ras-GTP levels. Expression of a constitutively activated MEK rescued tumor cells from *Grb10*-mediated reduction in colony formation. These studies reveal that *Grb10* loss can occur during *in vivo* tumorigenesis, with a functional consequence in untransformed primary cells. In tumors, *Grb10* loss independently promotes Ras pathway hyperactivation, which promotes hyperproliferation, an early feature of tumor development. In the context of a robust *Nf1* mutant mouse model of cancer this work identifies a novel role for an imprinted gene in tumorigenesis.

## Author Summary

Cancer-causing mutations typically involve either allele inherited from parents, and the parental source of a mutant allele is not known to influence the cancer phenotype. Imprinted genes are a class of genes whose expression is determined by a specific parental allele, either maternally or paternally derived. Thus, in contrast to most genes, the pattern of inheritance (maternal or paternal-derived) strongly influences the expression of an imprinted gene. Furthermore, imprinted genes can be differentially expressed in different tissue types. This work identifies a novel link between cancer and *Grb10*, an imprinted gene involved in organismal metabolism and growth. In our mouse model of radiation-induced tumors, we found monoallelic *Grb10* gene loss involving the parental allele responsible for protein expression. Tumors harboring genetic loss of the expressed *Grb10* allele showed absent transcript and total protein levels, despite an intact remaining wildtype *Grb10* allele identified by sequencing. When restored, Grb10 suppressed tumor growth by down-regulating Ras signaling. This work demonstrates a new role for an imprinted gene in tumor formation, and shows that *Grb10* functions to negatively regulate Ras signaling and suppress hyperproliferation.

## Introduction

Diverse types of somatic genetic alterations occur in cancers and play important roles in pathogenesis. A common cancer-promoting mechanism is the homozygous loss of a tumor suppressor gene, for example *Tp53* [1]. Classically, loss of tumor suppressor genes requires bi-allelic loss or inactivation, conforming to Knudsen’s two-hit hypothesis.

Tumor-promoting somatic mutations involve either allele, and the parental source of a mutant allele is not known to influence the cancer phenotype. A small fraction of genes, known as imprinted genes, are characterized by monoallelic expression from a single parental allele [2]. Heterozygous loss of the expressed parental allele produces a functionally nullizygous state [3]. Thus, the imprinting mechanism modulates gene expression in a manner that defies Mendelian predictions. To date, imprinted genes are not known to have a role in promoting the development of malignancies.

The tumor suppressor *NF1* gene, and its conserved murine homologue *Nf1*, encode the neurofibromin protein, which is ubiquitously expressed in mammalian cells and necessary for development [4]. Germline heterozygosity for *NF1* causes Neurofibromatosis I (NF1), an autosomal-dominant inherited disease with an incidence of 1 in 3000 live-births [5]. The development of benign and malignant neoplasms, typically during childhood, is a well-recognized feature of Neurofibromatosis I [5]. Furthermore, tumor genome analyses of diverse cancers have identified *NF1* mutations in sporadic but lethal cancers arising in adults, such as malignant brain tumors, ovarian cancers, and lung cancers [6–9].

The *NF1* gene encodes the neurofibromin protein, which functions as a Ras GTPase activating protein (GAP) [10], and loss of neurofibromin promotes hyperactivation of Ras signaling [11]. Oncogenic, constitutively activated Ras is frequently found in human cancers [12] and has been shown to play a causal role in tumor formation in many genetic models [13]. Although neurofibromin is a tumor suppressor protein, *NF1* loss alone is not sufficient to promote tumorigenesis. *NF1*-mediated tumorigenesis may thus require additional mechanisms to pathologically dysregulate Ras signaling, and as a consequence, additional therapeutically actionable steps may exist for inhibiting Ras signaling in the *NF1* null context.

To identify novel mutations and mechanisms that promote tumorigenesis with *Nf1* loss, we mutagenized mice heterozygous for *Nf1* with fractionated ionizing radiation [14,15]. These mouse models recapitulate clinical second malignant neoplasm (SMN) induction observed in NF1 individuals, and provide a novel approach for identifying the molecules cooperating in this process. Ionizing radiation exposure induces mutations, some of which may cooperate with *Nf1* heterozygosity to promote tumorigenesis. Mutagenizing *Nf1* ^*+/-*^ and wildtype mice with ionizing radiation generated diverse malignancies [14,15] from which we generated a unique panel of mouse tumor cell lines. Expression analysis of these lines revealed decreased Growth factor receptor bound protein 10 (Grb10) mRNA in *Nf1* null tumor cell lines compared to controls.

Grb10 is an adaptor protein that interacts with multiple receptor tyrosine kinases (RTK) [16,17]. Grb10 possesses a plekstrin homology (PH) domain, a Ras-Association (RA) domain, and a C-terminal Src homology 2 domain (SH2) [18], and associates with the insulin receptor, insulin-like growth factor receptor and epidermal growth factor receptor with variable affinities [19,20]. Grb10 is described to interact with proteins functioning downstream of RTKs such as Raf1 and MEK1 although the biological significance of these interactions are unclear [19]. Grb10 is also linked to Ras signaling through mTORC1 (mammalian target of rapamycin complex 1), which can phosphorylate Grb10 and regulate its levels by influencing protein stability [21,22].

Although biochemical evidence position Grb10 at multiple nodes in RTK signaling, questions persist concerning Grb10’s basic functions. In cell-based studies utilizing cultured fibroblasts, Grb10 promotes cell proliferation and survival [23]. *In vivo* evidence, however, indicate that Grb10 is an important *negative* modulator of proliferation and growth in tissues. Overexpression of *Grb10* in transgenic mice results in growth retardation and insulin resistance [24,25]. Conversely, *in vivo* loss of *Grb10* in mouse models increases animal size due to hyperproliferation of peripheral tissues, although these animals have no apparent propensity to develop cancers [26]. Analysis of enlarged muscle in *Grb10*-deleted mice reveals increased myofiber number rather than size, a phenotype that is present at birth and maintained throughout adulthood [27]. Thus, *in vivo* data support a role for *Grb10* as a negative regulator of proliferation and RTK signaling. *Grb10* is not known to have a role in tumor suppression, although *Grb10* expression is reduced in a wide range of human cancers [21].

*Grb10* is an imprinted gene in human and mice [3]. In the mouse, *Grb10* is expressed from the maternal allele in non-central nervous system (CNS) tissues [3]. Interestingly, radiation-induced tumors from our models all occur in non-CNS tissues, where *Grb10* is expressed from the maternal allele. Analysis of maternal and paternal-specific genetic polymorphisms established that the maternally expressed *Grb10* gene was lost in *cis* with wildtype *Nf1* in most radiation-induced tumors, providing a genetic mechanism for functional *Grb10* nullizygosity in tumors. Functionally, restoring Grb10 protein in *Nf1* null tumors suppressed tumor growth in a MAPK-dependent mechanism. Conversely, *Grb10* silencing promotes Ras signaling in and hyperproliferation of MEFs. This effect was *Nf1*-independent, although the most profound increase in Ras pathway activation occurred when both *Grb10* and *Nf1* were silenced. In human cancers, we found evidence for co-loss of neurofibromin and *Grb10* expression in human glioblastoma, a tumor type in which *NF1* is among the most significantly mutated genes [6]. Human tumor sequencing databases reveal that the *Grb10* and *NF1* genes can be co-mutated in diverse tumor histologies. In summary, this work identifies a role for an imprinted gene in promoting central features of tumorigenesis. In this context, we show that *Grb10* is a negative regulator of Ras signaling, and contributes significantly to Ras dysregulation in the setting of *Nf1*-mediated tumorigenesis. These findings demonstrate a previously undescribed role for an imprinted gene in disease and suggest that mutations in imprinted genes should be considered with regard to parental origin.

## Results

### *Grb10* expression is lost in *Nf1*-null tumors

**Fig 1:**
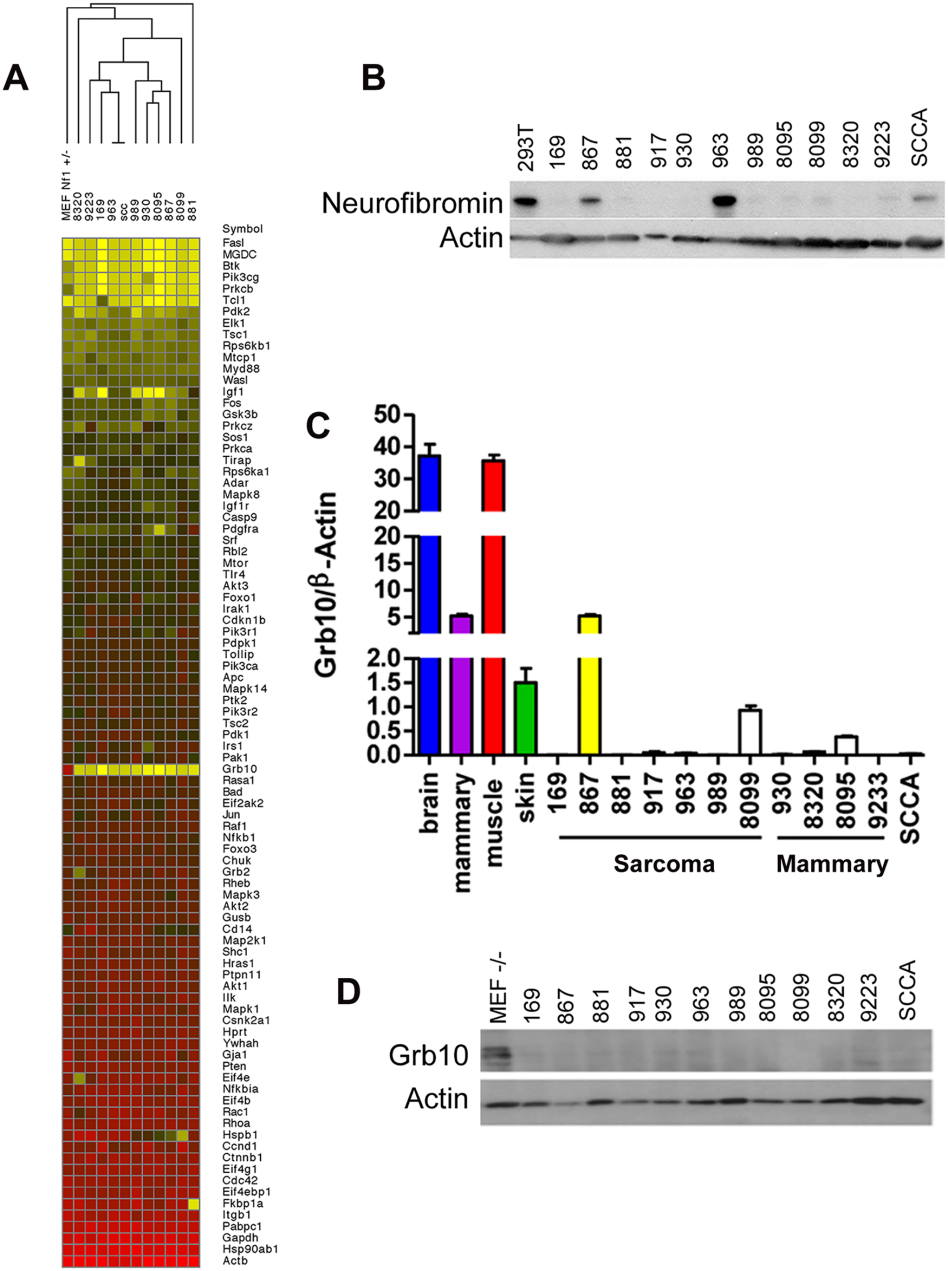
*Grb10* expression is reduced in *Nf1* mutant tumor cell lines. A. *Grb10* underexpression in tumor cell lines (SCCA is a squamous cell carcinoma cell line that arose in an irradiated *Nf1* ^*+/-*^ mouse). A heatmap representing data from a Rfdcdfuiop\saaa vb Qiagen PI3K Array comparing expression of PI3K-pathway related transcripts among tumor cell lines and *Nf1* ^*+/-*^ MEFs as control. *Grb10* expression is reduced in tumors compared to MEF control (yellow area at mid-column). B. Western blotting for neurofibromin protein and actin control. C. QPCR analysis of *Grb10* mRNA normalized to β-Actin shows significant decrease of Grb10 levels in tumor lines derived from *Nf1* ^*+/-*^ mice compared to normal adult tissue controls. D. Western blotting for Grb10 in corresponding tumor cell lysates shows uniformly reduced Grb10 protein levels as compared to MEF controls.

In earlier work, we mutagenized *Nf1* ^*+/*^ *-* and control wildtype mice to model second malignant neoplasms, severe complications that individuals with the NF1 syndrome develop can develop after radiotherapy [15]. C57Bl/6/129Sv *Nf1* ^*+/-*^ mice exposed to focal, fractionated ionizing radiation developed diverse malignancies, including soft tissue sarcomas, mammary carcinomas and squamous cell carcinomas. We established multiple tumor cell lines from primary radiation-induced tumors, two arising from wildtype mice (cell lines 867 and 963) and ten arising from *Nf1* ^*+/-*^ mice [14,15]. Using single nucleotide polymorphism and microsatellite analysis as previously described [15], we found that tumors from mutagenized *Nf1* ^*+/-*^ mice commonly lose the wildtype *Nf1* allele, rendering these tumors null for *Nf1*. Tumor formation in irradiated *Nf1* ^*+/-*^ mice is driven by complete loss of *Nf1*, which is also a hallmark of tumor formation in NF1 patients [28] and thus represents an early and necessary event. To identify mechanisms that are commonly altered in *Nf1-* mediated tumorigenesis and might function as second events, we interrogated our tumor cell lines using a targeted expression array to compare expression of known PI3K pathway regulators and effectors amongst our tumor lines (Fig 1A). This analysis identified *Grb10* expression as most uniformly reduced in tumors as compared to control untransformed *Nf1* ^*+/-*^ MEFs. We then confirmed by immunoblotting that loss of *Grb10* expression observed in Fig 1A was not neurofibromin-dependent (Fig 1B). Using quantitative PCR we independently verified *Grb10* expression in wildtype and *Nf1* mutant tumor cell lines relative to controls, and found that *Grb10* expression varied among multiple postnatal organs included as controls (Fig 1C), with greatest expression in brain and muscle as previously described [29]. In addition to brain and muscle, mammary and skin tissues were included as controls to represent the tissue types in which tumors originated (mammary carcinoma, sarcoma and squamous cell carcinoma). *Grb10* expression in 11 of 12 cell lines derived from our mouse models (the 867 cell line being the sole exception) was significantly reduced or undetectable (Fig 1C). Western blotting demonstrated that Grb10 protein was undetectable in all 12 tumor cell lines, including the 867 cell line (Fig 1D).

Grb10 belongs to a family of proteins that include Grb2, Grb7, and Grb14, whose members share protein domains but whose functions are not well-defined. To examine whether loss of *Grb10* expression might result in compensatory overexpression of these related Grb family members, we performed quantitative PCR to determine whether *Grb2*, *Grb7* or *Grb14* transcript levels increased when *Grb10* expression was reduced or lost. Similar to *Grb10*, *Grb2*, *Grb7* and *Grb14* expression varied among different control organs examined, however none were overexpressed in any tumor cell line compared to control tissues, arguing against compensatory overexpression of Grb family members in response to reduced *Grb10* expression (S1 Fig).

### The imprinted *Grb10* allele is lost *in cis* with the wildtype *Nf1* and *Trp53* alleles in tumors from *Nf1* ^*+/-*^ mice

The *Grb10* gene, initially named *Maternally expressed gene 1* (*Meg1*), is imprinted in mice and humans [26,30–32]. *Grb10* expression from either the paternal or maternal allele segregates between the central nervous system (CNS) and non-CNS tissues [3]. In mice, paternally-derived *Grb10* is expressed exclusively in the CNS, while maternally-derived *Grb10* is expressed in remaining non-CNS tissues, such as muscles [3,33]. This pattern of tissue restriction is conserved in humans (CNS versus non-CNS), although the allelic contribution is reversed (i.e. maternal expression in the CNS). Because *Grb10* expression is imprinted, monoallelic deletion, depending on the affected cell type, can functionally reproduce homozygous allelic loss [34]. Consistent with this, mouse models with targeted loss of either the paternal or maternal *Grb10* allele demonstrate loss of expression in discrete tissue compartments and non-overlapping phenotypes [3].

*Grb10* expression is reduced in a variety of human cancers [21], although the underlying mechanism for this reduction has not been defined. Earlier data implicate *Grb10* as a candidate modifier gene cooperating with *Nf1* loss to promote tumors. Indeed, astrocytoma formation in mice after co-loss of *Nf1* and *Trp53* is strongly influenced by the parental origin of the mutant chromosome 11 [32,35], on which *Nf1*,*Trp53*, and *Grb10* genes reside. A mechanism for *Grb10* contributing to tumorigenesis either in human or mouse tumors is currently undefined.

**Fig 2:**
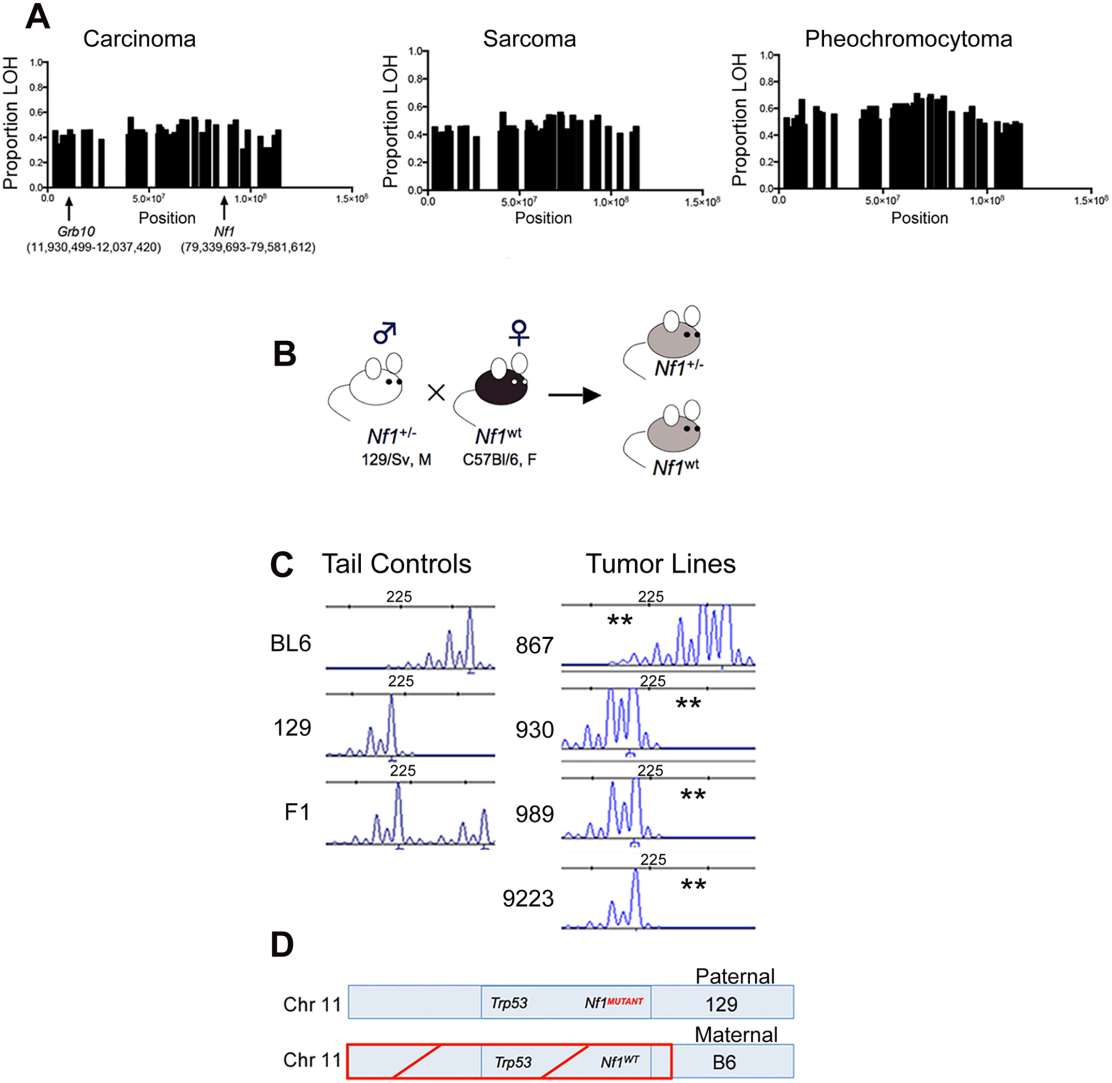
*Grb10* chromosomal position, inheritance, and loss in *Nf1* mutant mouse tumors. A. Proportion of tumors showing LOH along chromosome 11 in *Nf1* mutant tumors. (Carcinomas n = 26, sarcomas n = 26, pheochromocytomas n = 52) B. Breeding schema describing inheritance of mutant and wildtype *Nf1* genes. C. Microsatellites assessed by PCR-based fragment analysis to verify LOH in *Grb10* gene and identify parental allele involved (BL6 or 129). (**) indicates loss. Reduced C57Bl/6-derived peak in *Nf1* mutant cell lines 930, 989, 9223, indicating LOH of maternal C57Bl/6 allele, while cell line 867 has loss of the paternal 129-derived allele. D. Schematic of predominant pattern of *Grb10* and *Nf1* loss.

We have shown previously that the *Nf1* gene and the *Trp53* genes on chromosome 11 are co-lost in tumors arising in *Nf1* ^*+/-*^ mice [15]. To determine whether there was a similar genetic basis for *Grb10* loss in our tumors, we exploited the F1 background of our model to analyze for loss of heterozygosity (LOH) in *Grb10*. Tumors from *Nf1* mutant mice were analyzed using Illumina Medium density array-based SNP genotyping to assess for LOH along chromosome 11. Consistent with our earlier findings [15], LOH occurred in chromosomal regions spanning across *Nf1* (Fig 2A, S1 Table), and this pattern of chromosomal loss was present in all three major tumor histologies arising from our mouse models (carcinoma, sarcoma and pheochromocytoma). Interestingly, LOH on chromosome 11 extended beyond the *Nf1* locus to involve most of the chromosome, raising the possibility that a gene centromeric to the *Nf1* gene drives this loss. The extent of LOH on chromosome 11 was similar between carcinomas, sarcomas, and pheochromocytomas arising in *Nf1* mutant mice. Interestingly, the *Grb10* gene localizes to a region involved with LOH (Fig 2A), suggesting that genetic loss of the expressed *Grb10* allele may underlie the absence of *Grb10* transcripts in *Nf1* mutant tumor cell lines (Fig 1).

To orthogonally validate a genetic mechanism for the loss of *Grb10* expression in our tumors we performed microsatellite-based loss of heterozygosity (LOH) analysis. The design of our mouse models employed a fixed breeding schema in which the wildtype *Nf1* allele was always maternally-derived (Fig 2B). Our mouse models also utilize an F1 background so that parental alleles can be distinguished at specific loci (Fig 2C). Because the tumors isolated from our mouse models are non-CNS tumors, *Grb10* expression would be derived from the maternal *Grb10* allele. Analysis of primary tumor samples identified 19 tumors from *Nf1* ^*+/-*^ mice in which *Nf1* and *Grb10* status could be co-determined. 17 of these 19 (89%) demonstrated LOH of the maternal *Grb10* allele, and 2/19 (11%) had LOH of the paternal allele. Among the 17 tumors demonstrating LOH of the maternal *Grb10* allele, in 12/17 (71%) loss occurred in *cis* with the wildtype *Nf1* gene. Loss of the paternally-derived *Grb10* allele (in *trans*) with *Nf1* and *Trp53* alleles occurred in only 1/19 tumors, and 1/19 tumors showed LOH of paternal *Grb10* without *Nf1* co-loss. In summary, this analysis independently confirmed *Grb10* LOH in the majority of tumors from *Nf1* ^*+/-*^ mice and localized loss to involve the maternal allele.

Among radiation-induced tumors from wildtype mice, *Nf1* and *Grb10* status was determined in 7 tumors. Four of 7 (57%) demonstrated maternal *Grb10* loss, and the remaining 3 (43%) showed loss of the paternal allele. Of the 4 tumors with maternal *Grb10* loss, 2 (50%) had loss of *Nf1* (1 in *cis* and 1 in *trans*), and in the remaining 2 tumors LOH of *Nf1* was not detected (heterozygosity was intact).

Taken together, these data localize the majority of *Grb10* loss in both *Nf1* mutant and wildtype tumors to the maternal allele, on which the wildtype *Nf1* and *Trp53* alleles are also lost in *Nf1* mutant tumors (Fig 2C and 2D). The co-loss of all three of these genes in *cis* from chromosome 11 suggests a common genetic mechanism or event driving these losses.

We also performed *Grb10* microsatellite analysis on our tumor cell lines (the corresponding primary tumors from which these lines are derived were analyzed above) shown in Fig 1. All lines except for 867 demonstrated LOH of the maternally-derived *Grb10* allele (in *cis* with the wildtype *Nf1* allele). The 867 tumor cell line, which was established from a tumor arising in an irradiated wildtype mouse and expresses *Grb10* transcript levels similar to normal tissues (Fig 1), demonstrates loss of the paternal *Grb10* allele (Fig 2C). 867 is a sarcoma cell line, and loss of the silenced paternal allele (with retention of the maternal allele) likely explains why this tumor has detectable *Grb10* transcripts. However, immunoblotting for total Grb10 protein demonstrates absence of Grb10 protein in all tumor cell lines, including 867, suggesting that *Grb10* loss in the 867 cell line results from alternative, possibly post-translational mechanisms. We sequenced the *Grb10* exons in all our tumor cell lines, which revealed no mutations in the remaining paternal *Grb10* allele (sequence data from representative cell lines 881, 963 and 989 are archived at http://www.ebi.ac.uk/ena/browse) and is consistent with preferential loss of the maternal *Grb10* allele resulting in functional nullizygosity (Fig 2D). These data support the idea that genetically-mediated loss of *Grb10* expression suggests a role for *Grb10* in suppressing tumorigenesis.

### Restoration of *Grb10* expression in *Grb10* null tumor cell lines reduces tumorigenicity *in vitro* and suppresses Ras signaling

**Fig 3:**
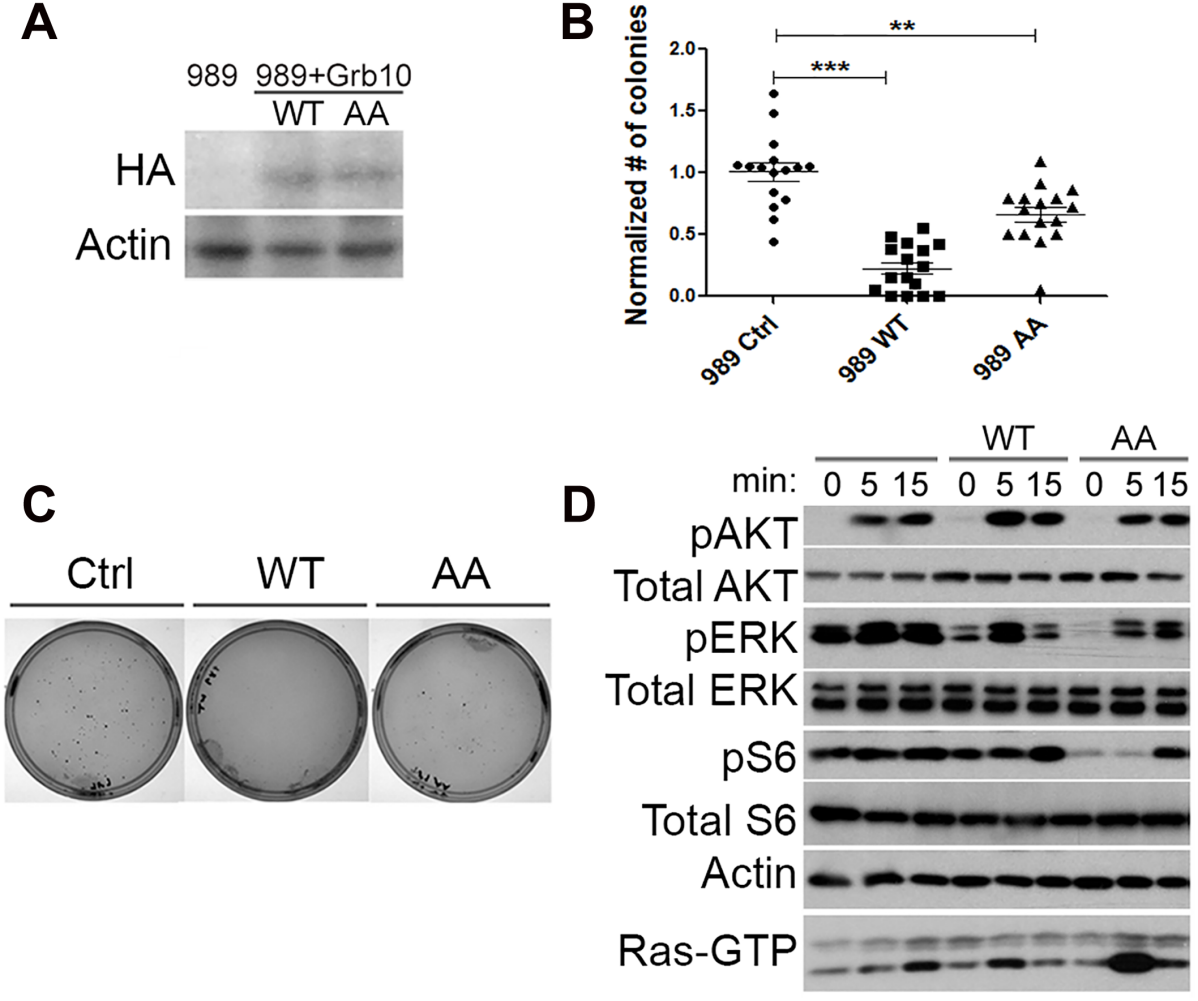
*Grb10* expression reduces soft agar colony formation by *Nf1* mutant tumor cell line. A. Retro-virally expressed HA-Grb10 protein in 989 tumor cells was identified by immunoblotting for the HA tag. B. Soft agar colony formation assay quantified for each cell line. Data from 4 independent experiments, each with replicates of 4. (Student’s t-test, *** p<0.001, *p< 0.05) C. Photographs of representative plate stained for colonies from each group. D. 989 tumor cells expressing either wildtype or mutant Grb10 were serum starved for 18 hours, then stimulated with insulin (150 nM). Whole cell lysates of tumor cells were collected at 0, 5 and 15 minutes after insulin was added, then assessed by immunoblotting for phospho-specific antibodies against activated Ras effectors Akt Ser 473, ERK 42/44 Thr 202/Tyr204, and S6 Ser235/236. Corresponding Ras-GTP pull-down is shown on bottom line.

As a negative regulator of growth factor signaling, *Grb10* restoration is predicted to reduce Ras signaling through the downstream PI3K and MAPK pathways. To test whether *Grb10* restoration altered tumor formation or Ras signaling, retrovirus was used to stably express either wildtype or mutant Grb10 protein (AA) in multiple *Nf1* mutant tumor cell lines. The Grb10 AA mutant bears mutations of the mTORC1 phosphorylation sites Serine 501 and Serine 503 to alanines (AA) [21]. Although serine phosphorylation has been proposed to increase protein stability [21], the level of mutant *Grb10* expression was similar to that of wildtype Grb10 in our cell lines (Fig 3A).

Restoring wildtype *Grb10* significantly reduced soft agar colony formation by the *Nf1* null cell lines 989 (shown in Fig 3B and 3C) compared to control and mutant *Grb10*. To explore the possible mechanism of Grb10-mediated inhibition of colony formation we assessed signaling downstream of Ras in these cells. Cells expressing wildtype *Grb10* demonstrated reduced phosphorylated ERK after serum starvation as well as after insulin stimulation compared to control cells (Fig 3D). These data indicate that restoration of *Grb10* can profoundly reduce colony formation, attenuate pro-oncogenic signaling, and decrease basal proliferation in *Grb10* deficient tumor cell lines. These data thus identify the loss of *Grb10* as a mechanism that supports a significant growth advantage that may be selected for during tumorigenesis.

Expression of the *Grb10* AA mutant resulted in colony formation intermediate between wildtype *Grb10* and control, suggesting that the mutated Serine 501/503 residues produce partial loss of function.

Mutant *Grb10* expression had unexpected effects on signaling, characterized by suppression of MAPK pathway signaling after serum starvation as well as after stimulation with insulin, compared to control. Phosphorylated Akt levels were similar to control, but basal phosphorylated S6 levels were reduced and stimulation was delayed.

Phosphorylation of Akt after insulin exposure was similar in amplitude and duration among the three cell lines. Ras-GTP levels in cells expressing wildtype *Grb10* paralleled the phosphorylation of ERK (Fig 3D). The difference in ERK1/2 phosphorylation kinetics after insulin exposure between the wildtype and mutant *Grb10*, coupled with the colony formation data in Fig 3B, suggests that the mutant *Grb10*’s less effective inhibition of MAPK signaling may underlie the more modest suppression of colony formation observed with this variant.

### *Grb10* silencing promotes cell proliferation in untransformed wildtype and *Nf1* mutant MEFs

Hyperproliferation is a hallmark feature of transformation. Inappropriate cell proliferation occurs early in tumor development, and increasing cell proliferation rates typically correlate with tumor progression and aggressive clinical behavior [36]. Oncogenic activation of Ras signaling pathways in normal cells triggers compensatory mechanisms and negative feedback to suppress inappropriate proliferation, a phenomenon known as oncogene-induced senescence [37]. The feedback mechanisms underlying this protective response are not well-defined, but require p53 and Rb [37,38].

**Fig 4:**
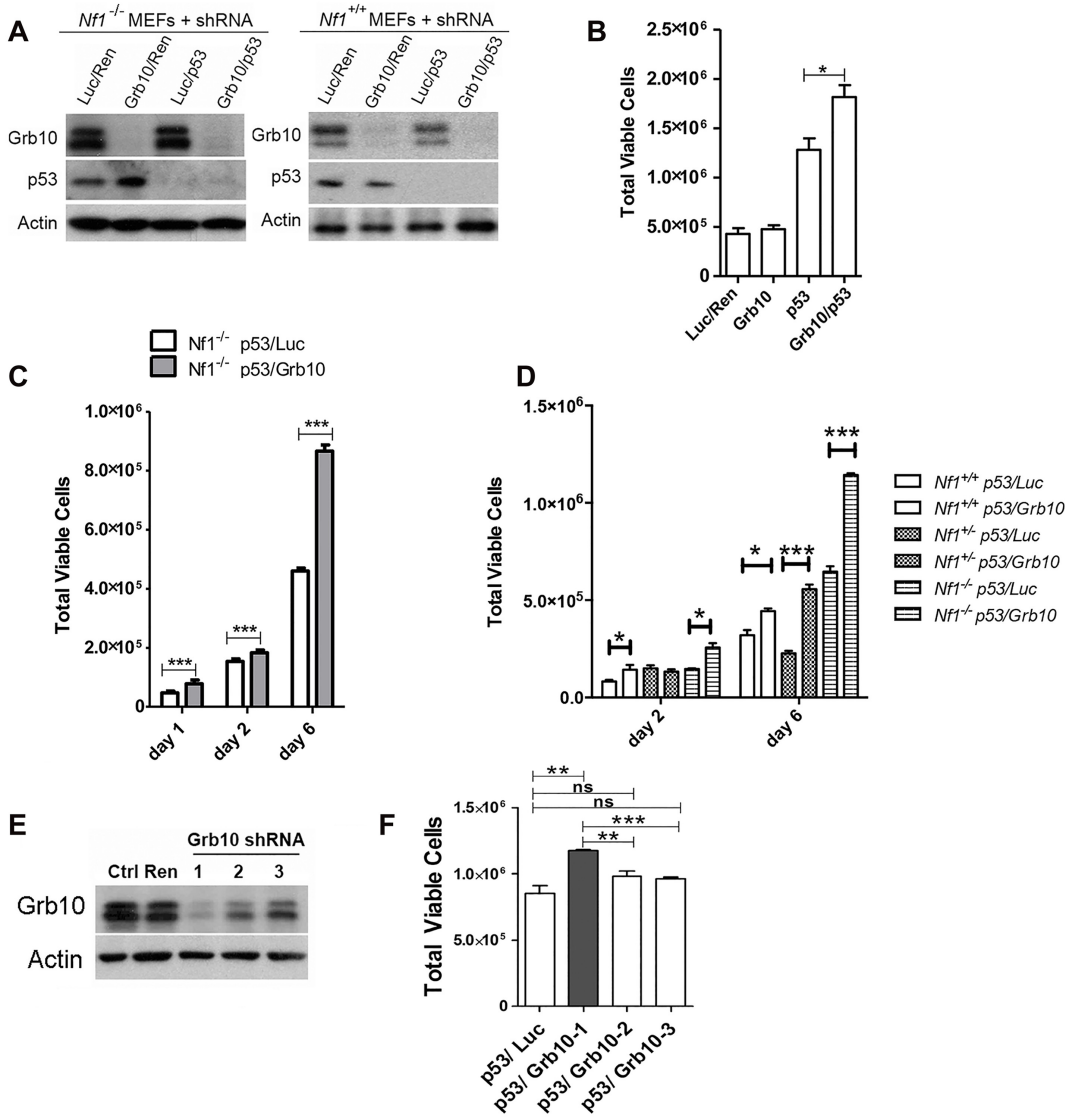
*Grb10* knockdown in MEFs increases cell proliferation. *p53* and *Grb10* expression in MEFs was silenced using lenti-virally expressed shRNAs. A. Immunoblot showing shRNA-mediated silencing of p53 and/or Grb10 in wildtype and *Nf1* null MEFs. Ren—control shRNA against Renilla, Luc—control shRNA against Luciferase. B. Day 2 post plating. Cell counts performed in triplicate. t-test,*p = 0.03. C. *Nf1 44* ^*-/-*^ MEFs in which shRNA against p53 and Luciferase (control) or p53 and Grb10, are expressed. ***p<0.001. D. Increased cell proliferation (day 6) mediated by *Grb10* loss is genotype-independent (t-test, *p<0.05, ***p<0.001). B, C, D are three independent experiments/transduced sets of cells. E. Three different shRNA against Grb10 were stably expressed in MEFs. Lysates from cells expressing each of the shRNAs and control lysates were immunoblotted for total Grb10 protein and actin. F. Proliferation among MEFs expressing each of Grb10 shRNA are compared (Student’s t-test, ns—not significant, ** p<0.001, ***p<0.0001).

*Grb10* expression is reduced in diverse human tumors [21], but the timing of *Grb10* loss in tumorigenesis and whether *Grb10* loss mediates early features of tumorigenesis are completely unknown. If *Grb10* loss occurred as an important event early in tumorigenesis, a likely consequence could be increased cell proliferation, the basis for pre-malignant hyperplasia. Interestingly, the pattern of LOH characterized by *Grb10* loss *in cis* with *Nf1* and *Trp53* in our tumors is suggestive of a chromosomal break that produced this extensive chromosomal loss early in tumor development. To replicate this genetic signature as an early event in untransformed cells we used lentivirus to stably express shRNA targeting *Trp53* alone or with shRNA targeting *Grb10* in *Nf1* null MEFs (Fig 4A). Silencing *Grb10* alone produced no significant change in basal MEF proliferation, as measured by cell counts (Fig 4B). Silencing of *Trp53* alone, however, significantly increased cell proliferation rates (Fig 4B), and co-silencing of *Trp53* and *Grb10* further significantly increased cell proliferation by *Nf1* null MEFs over that associated with *Trp53* silencing alone (Fig 4B). Hyperproliferation associated with co-silencing was sustained over several days (Fig 4C), indicating that cells failed to invoke compensatory mechanisms to suppress proliferation. We then tested whether the MEF hyperproliferation mediated by *Grb10* loss was influenced by *Nf1* status, and silenced *Trp53* with and without *Grb10* in wildtype, *Nf1* ^*+/-*^ and *Nf1* ^*-/-*^ MEFs (Fig 4D). Adding *Grb10* silencing to *Trp53* silencing further increased cell proliferation by day 6 for all genotypes (Fig 4D), indicating that this effect does not require *Nf1* loss. However, co-loss of *Nf1* and *Grb10* was associated with greater cell proliferation as compared to proliferation with silencing of each alone; thus over time, *Grb10* loss produced the greatest relative increase in cell proliferation when combined with *Nf1* loss, either heterozygous or homozygous (Fig 4D). To further determine whether the increased cell numbers associated with *Grb10* silencing reflect proliferation as opposed to altered cell loss, we assessed the proliferative index by BrdU labeling (S2 Fig). This data indicates that *Grb10* silencing in MEFs significantly increases BrdU incorporation, consistent with *Grb10* silencing promoting proliferation.

We tested multiple unique shRNAs against *Grb10* and expressed each of these in *Nf1* ^*-/-*^ MEFs, then assessed the efficacy of silencing by immunoblotting and the quantifying the effect on proliferation. Our panel of *Grb10* shRNAs achieved variable degrees of silencing (Fig 4E), and cell hyperproliferation correlated with the degree of *Grb10* silencing, with significantly increased cell proliferation requiring near-complete depletion of Grb10 protein levels (Fig 4F).

### *Grb10* silencing increases Ras signaling in untransformed MEFs independent of genotype

**Fig 5:**
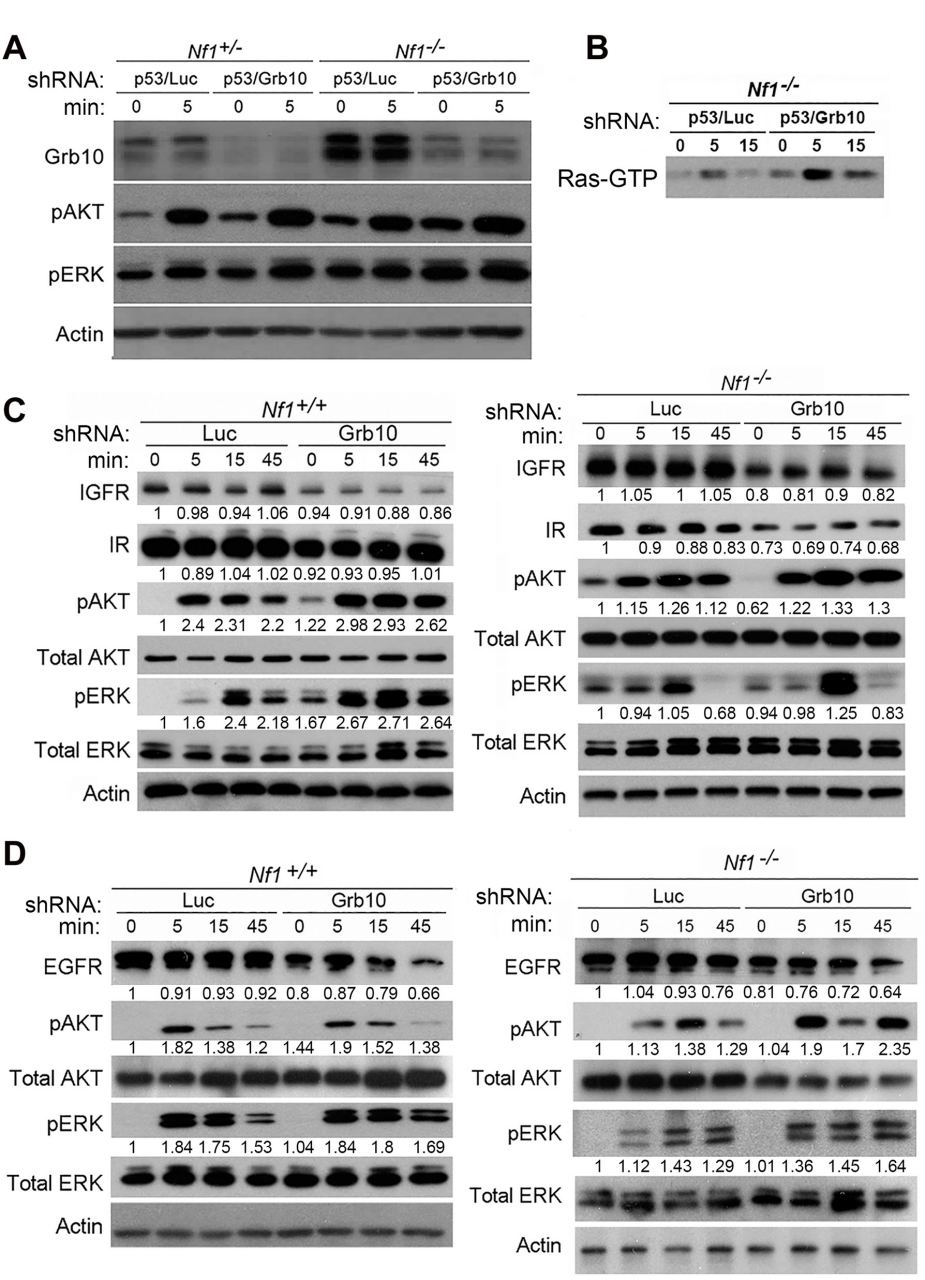
*Grb10* silencing in MEFs increases Ras signaling. A. *Nf1* ^*+/-*^ and *Nf1* ^*-/-*^ MEFs expressing shRNA against *Trp53* and shRNA against *Luciferase* (control) or *Grb10* were serum starved overnight, then stimulated with insulin. Whole cell lysates were collected at shown timepoints and immunoblotting performed. B. Ras-GTP pulldown MEFs serum starved overnight, then stimulated with insulin. C/D. Wildtype and *Nf1* ^*-/-*^ MEFs expressing shRNA against Luciferase (control) or *Grb10* were serum starved overnight, then stimulated with insulin (panel C) or EGF (panel D). Whole cell lysates were collected at shown time points and immunoblotted.

*Grb10* restoration in *Nf1* mutant tumor cells reduced colony formation, proliferation, and suppressed Ras signaling, suggesting Grb10’s tumor suppressive effects are mediated by modulating Ras pathway activation. As shown above, *Grb10* silencing increased the proliferation of untransformed cells. To determine whether MEF hyperproliferation after *Grb10* silencing, similar to tumor cells, involved Grb10-dependent effects on Ras signaling, we used phospho-specific immunoblotting to assess Ras effector activation in the presence or absence of *Grb10* silencing. After stably silencing *Grb10* and *Trp53* in *Nf1* ^*+/-*^ or *Nf1* ^*-/-*^ MEFs, cells were serum starved for 18 hours, stimulated with insulin (75 nM), and lysates were collected at 0 and 5 minutes. Immunoblotting for total Grb10 protein confirmed the silencing achieved with shRNA, and interestingly revealed that basal Grb10 protein levels increase when *Nf1* is fully lost (Fig 5A), suggesting that Grb10 can be dynamically modulated to negatively regulate Ras signaling. Immunoblotting with phospho-specific antibodies showed that silencing *Grb10* increased phosphorylated Akt and ERK levels after serum starvation (Fig 5A, comparing lanes 1 to 3, and lanes 5 to 7) as well as after stimulation, with the greatest absolute levels of phosphorylated Akt and ERK attained in *Nf1* ^*-/-*^ MEFs after *Grb10* silencing (Fig 5A). Paralleling the effects on Ras effectors, *Grb10* silencing increased Ras-GTP levels in serum starved *Nf1* mutant MEFs stimulated with insulin (Fig 5B). Assessing Akt and ERK phosphorylation at later timepoints revealed that *Grb10* silencing was associated with hyperactivation of Akt and ERK at 45 minutes post stimulation with either insulin or EGF (Fig 5C and 5D). Ras signaling hyperactivation mediated by *Grb10* loss was independent of *Trp53* silencing, as MEFs expressing shRNA against *Grb10* only demonstrated increased Akt and ERK phosphorylation after insulin (Fig 5C) and EGF (Fig 5D) stimulation.

To determine whether *Grb10* silencing altered levels of the receptors through which stimulation is mediated, we assessed total protein levels of Insulin Receptor (IR), Insulin-like Growth Factor Receptor (IGFR) and Epidermal Growth Factor Receptor (EGFR) in cells after *Grb10* silencing (Fig 5C and 5D). *Grb10* silencing was associated with decreased total levels of IR, IGFR and EGFR (Fig 5C), indicating a multi-receptor downregulation in response to *Grb10* silencing. Furthermore, *Grb10* silencing in both wildtype and *Nf1* null MEFs was associated with a reduction of total EGFR levels at 45 minutes post-stimulation (Fig 5D). These reductions in total receptor levels would be predicted to reduce proliferation and growth signaling in response to insulin or Epidermal Growth Factor. Although *Grb10* silencing reduced total levels of insulin receptor, phosphorylated insulin receptor levels were increased in the setting of serum starvation as well as after insulin stimulation in *Grb10*-silenced MEFs (wildtype and *Nf1* ^*-/-*^) (S3 Fig), suggesting that Grb10 protein suppresses receptor autophosphorylation and degradation. Despite this, *Grb10* loss, in an *Nf1*-independent manner, renders cells resistant to downregulation of Ras signaling by serum starvation, and increases activation of Ras signaling in response to insulin and EGF. Together, these data indicate that *Grb10* modulates Ras signaling in untransformed cells as well as tumor cells, and that loss of *Grb10* in untransformed cells increases the magnitude and duration of Ras effector activation despite reduced insulin receptor levels.

### Expression of constitutively activated MEK abrogates the effect of wildtype *Grb10* expression on colony formation

**Fig 6:**
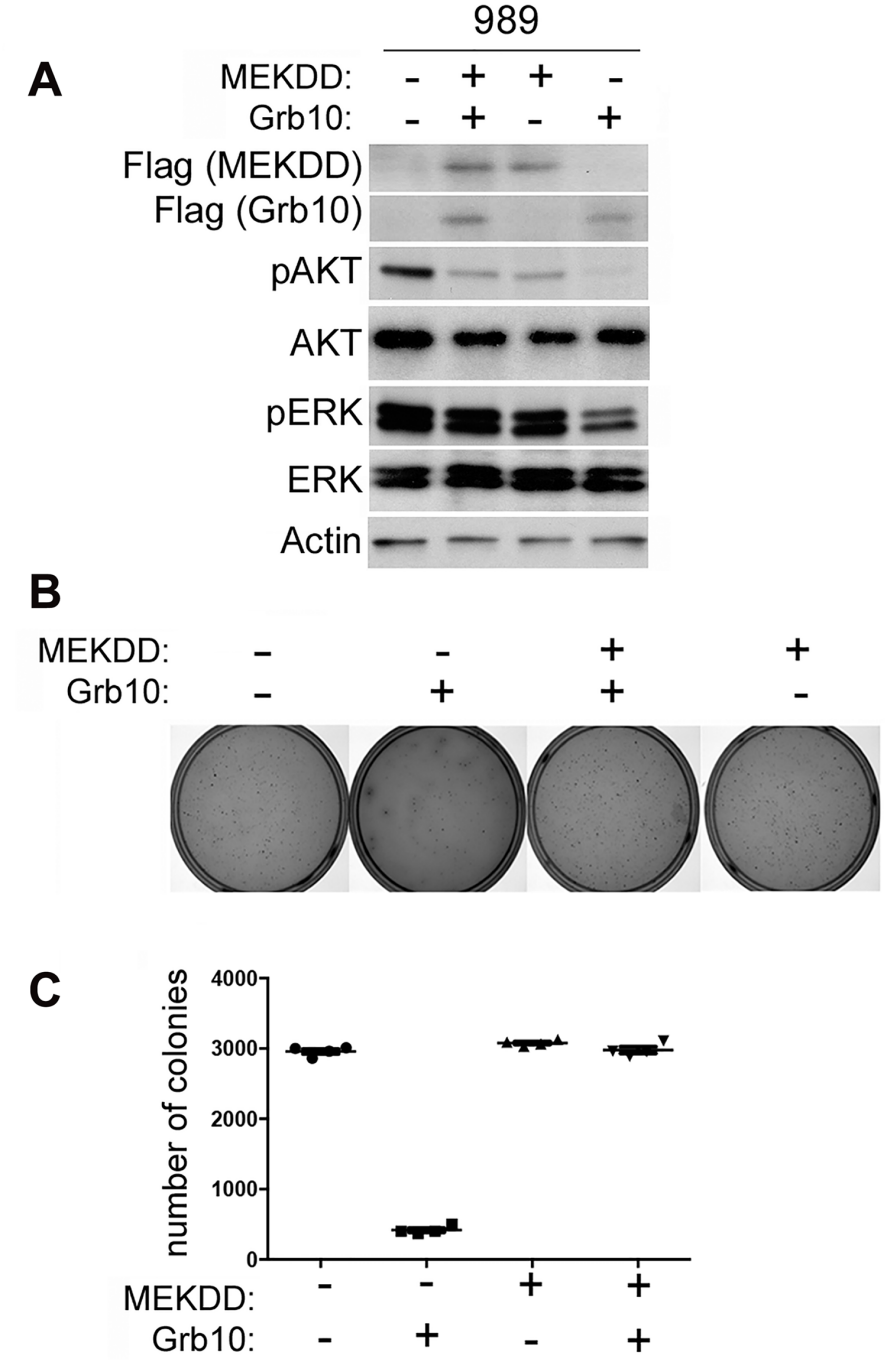
Expression of constitutively activated MEK rescues tumor cells from *Grb10* mediated suppression of colony formation. A. Flag-tagged Grb10, MEK DD, or both were expressed in 989 tumor cells. Expression was confirmed by immunoblotting for Flag and phosphorylated Akt and ERK were visualized. Actin is shown as loading control. B/C. 989 tumor cells expressing Flag-tagged Grb10, MEK DD, or both, as shown in panel (A), were grown in soft agar colony formation assay. Photographs of representative plates stained for colonies (B) and quantitation of colony formation (C) are shown.

Although Grb10 levels influence both PI3K and MAPK signaling in untransformed MEFs, in our tumor lines MAPK pathway activation was suppressed by Grb10 restoration. To determine whether Grb10-mediated suppression of colony formation by tumor cells requires inhibition of MAPK signaling, we expressed a constitutively activated Flag tagged-MEK (MEK DD) [39] alone or with wildtype Grb10 in 989 tumor cells. We confirmed expression by immunoblotting (Fig 6A) and assessed phosphorylated Akt and ERK levels (Fig 6A). Restoring *Grb10* expression in 989 cells reduced phosphorylated Akt and ERK levels (Fig 6B). Expression of mutant MEK DD restored phosphorylated ERK levels but not phosphorylated Akt (Fig 6A). Comparing colony formation between the cell lines showed a significant reduction in colony formation by 989 tumor cells after *Grb10* expression as compared to control, which was rescued with co-expression of MEK DD with *Grb10* (Fig 6B and 6C).

### *Grb10* expression reduces the proliferation of mouse and human tumor cells in multiple oncogenic backgrounds

**Fig 7:**
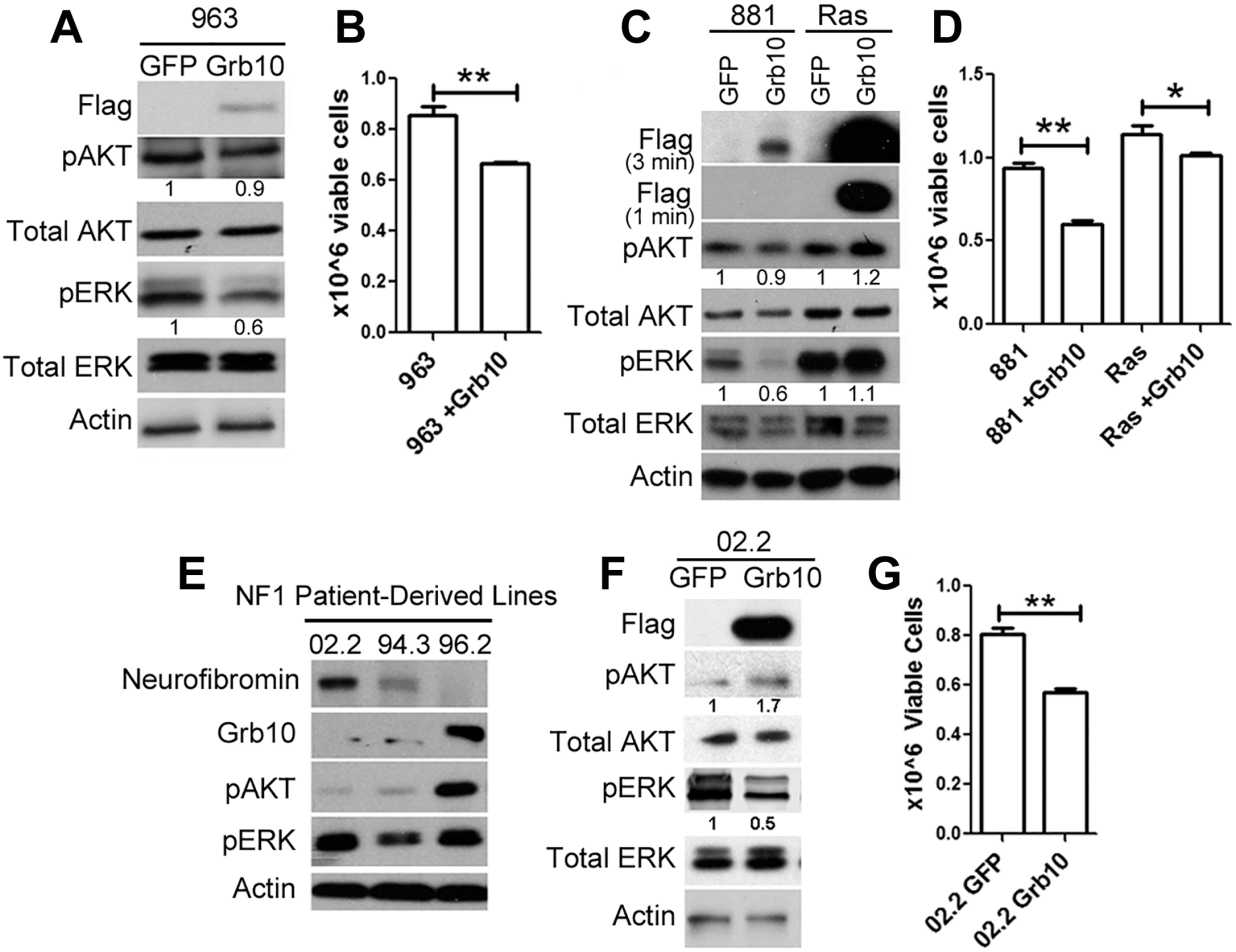
*Grb10* expression in diverse tumors suppresses proliferation. A. Wildtype *Grb10* or GFP was expressed in sarcoma line 963, which is wildtype for *Nf1*. *Grb10* restoration was confirmed by immunoblotting for the FLAG tag, and pAKT and pERK levels were assessed. B. Cell proliferation was assessed 4 days after plating and was reduced in 963 tumor cells after *Grb10* restoration (t-test, **p<0.01). C. Wildtype *Grb10* or GFP was expressed in ^*V16*^HRas-transformed human astrocytes and 881 cells for comparison. *Grb10* overexpression was confirmed by immunoblotting for Flag, and phosphorylated and total AKT and ERK were assessed. To visualize the markedly different levels of *Grb10* expression between the cell lines, two exposures (3 minutes and 1 minute) are shown. D. Cell proliferation was assessed 4 days after plating was significantly reduced in 881 (null for *Nf1*) and ^*V16*^HRas-transformed human astrocytes expressing *Grb10* (t-test, **p<0.01). E. The *NF1* mutant human tumor cell lines 02.2, 94.3, 96.2 were assessed for total Grb10 protein, and phosphorylated AKT and ERK by immunoblotting. F. GFP or FLAG-tagged wildtype *Grb10* was expressed in the human *NF1* mutant tumor cell line 02.2. Cell lysates were immunoblotted for the FLAG tag, phosphorylated AKT and ERK, total AKT and ERK, and Actin. G. 02.2 cells expressing either GFP or Grb10 were compared for differences in cell proliferation rates after 4 days in culture (t-test, **<0.01). The histogram is representative of 1 of 3 experiments.

Our data are consistent with Grb10 functioning to negatively regulate Ras activation, with *Grb10* loss resulting in downstream activation of PI3K and MAPK pathways paralleling Ras-GTP levels. This function had been identified in an *Nf1* mutant tumor cell line. To determine whether this effect in tumors was *Nf1* dependent, we expressed *Grb10* in the sarcoma line 963, which arose in a wildtype mouse and expresses neurofibromin protein. *Grb10* restoration significantly decreased pERK levels and proliferation in these tumor cells, indicating that Grb10-mediated growth suppression does not require *Nf1* loss (Fig 7A and 7B). We then tested whether *Grb10* over-expression suppresses Ras signaling in cells transformed by oncogenically-mutated Ras, a common mechanism for Ras pathway hyperactivation in tumors. We expressed Flag-tagged wildtype *Grb10* in human astrocytes transformed with retrovirus encoding ^V16^HRas [40], a mutant Ras that is constitutively activated. Flag-tagged wildtype *Grb10* was also expressed in the *Nf1* mutant/*Grb10* null mouse tumor cell line 881 (neurofibromin and Grb10 protein levels previously shown in Fig 1B and 1D) for comparison. *Grb10* expression was confirmed by immunoblotting, which showed that Ras-transformed human astrocytes demonstrated marked overexpression of *Grb10* compared to 881 tumor cells (Fig 7C). *Grb10*-overexpressing ^V16^HRas-transformed human astrocytes demonstrated sustained phosphorylated ERK, in contrast to the *Nf1* mutant tumor cell line 881, which similar to the 989 tumor cell line showed significantly reduced MAPK signaling and slightly reduced Akt phosphorylation (Fig 7C). We assessed cell proliferation to determine whether similar to the MEFs and *Nf1* mutant tumor cell lines *Grb10* expression suppressed cell proliferation of Ras-transformed cells. *Grb10* restoration significantly decreased proliferation by the *Nf1* null 881 tumor cells, and to a lesser extent, reduced cell proliferation in ^V16^HRas-transformed human astrocytes (Fig 7D). This modest effect in ^V16^HRas-transformed human astrocytes may reflect the known resistance of mutant Ras to modulation and may be mediated by *Grb10*’s effect on endogenous wildtype Ras present in these tumors cells. Together these results point to a mechanism of Grb10 action upstream of Ras activation.

We assessed human malignant peripheral nerve sheath tumors that arose in individuals with NF1 for total Grb10 protein levels. SNF02.2, SNF94.3 and SNF96.2 are established human MPNST cell lines harboring mutant *NF1* [41]. Two out of the 3 lines examined (lines 02.2 and 94.3) showed decreased levels of Grb10 protein (Fig 7E). Restoring *Grb10* expression in lines 02.2 reduced MAPK, but not PI3K activity, as measured by phosphorylated ERK and AKT, respectively (Fig 7F). *Grb10* restoration also decreased proliferation in line 02.2, mirroring the previous effects observed in the sarcoma line 989 from our mouse model (Fig 7G).

## Discussion

This work identified *Grb10* as genetically lost in most tumors developing in our *Nf1* mutant-based mouse model. Our subsequent functional studies show that *Grb10* loss promotes oncogenic signaling through hyperactivation of Ras signaling as well as the downstream MAPK pathway. The biochemical data are consistent with Grb10 protein functioning as a negative regulator of growth factor receptor signaling, and when lost with another negative regulator of Ras contributes to tumorigenesis. Although the initial identification of lost *Grb10* expression was made in our *Nf1* mutant-based mouse model of cancer, studies in human tumor cell lines as well as malignant cells generated from *Nf1* wildtype backgrounds suggest that *Grb10* loss may have tumor-promoting consequences in other oncogenic contexts.

This work sheds light on the cellular responses to *Grb10* loss, alone and when combined with *Nf1*. Total Grb10 protein levels increased in MEFs with *Nf1* loss alone. These findings indicate that Grb10 levels are modulated in response to alterations in Ras pathway control, suggesting that increased *Grb10* expression is a compensatory response to *Nf1* loss. However, this compensation is incomplete and the physiologic increases in Grb10 protein observed in *Nf1* null MEFs fail to functionally replace neurofibromin, as gauged by Ras pathway activation. This is evidenced by the fact that *Nf1* null MEFs demonstrate significantly increased Ras pathway activation compared to *Nf1* ^*+/-*^ MEFs (Fig 5A) despite increased *Grb10* expression. Our experiments employing shRNA in MEFs show that silencing *Grb10* increases Ras signaling in an *Nf1*-independent manner, and in an *Nf1* null context Ras activation can be increased further by *Grb10* silencing, and this is associated with a proliferative advantage exceeding that conferred by *Nf1* loss alone (Fig 4). Total Grb10 protein levels are rapidly modulated in other cellular contexts with physiologic consequences, for example adipocytes exposed to pharmacologic mimetics of cold stress [42]. We also found that *Grb10* silencing has upstream consequences at the level of membrane receptors. *Grb10* silencing was associated with decreased protein levels of multiple growth-promoting receptors, namely EGFR, IR and IGFR. Together, these data implicate Grb10 as a player in the dynamic modulation of receptor tyrosine kinases and the regulation of Ras signaling.

Grb10 is a substrate for mTORC [21,22], and in brown adipocytes, it was recently described that Grb10 functions in a feedback loop with the insulin receptor and raptor to inhibit mTORC1 signaling [42]. In that work, transient overexpression of wildtype *Grb10* in HEK293 cells was associated with decreased phosphorylation of S6Kinase after insulin exposure [42], an effect that was abrogated when mTORC1 phosphorylation sites on Grb10 were mutated to alanine. In our study, restoration of wildtype *Grb10* expression in *Nf1* mutant tumor cells lacking *Grb10* expression failed to suppress phosphorylation of S6Kinase upon exposure to insulin (Fig 3D). This difference between HEK293 cells and our *Nf1* mutant tumor cells may reflect the possibility that a Grb10-mediated negative feedback loop acting upon mTOR also requires fully intact Ras regulation, such as neurofibromin. Furthermore, *Grb10* has tissue-specific functions *in vivo*, and an additional possibility is that Grb10’s function in a transformed cell may be specific to both the cell of origin as well as the mutational background.

*Grb10* restoration in Ras-transformed human astrocytes decreased cell proliferation (Fig 7D), suggesting that even in cells expressing mutant Ras, Grb10 levels influence tumor growth. This finding does not invalidate the observation that *Nf1* mutant cells benefit from Grb10 loss. Thus, *Grb10* loss may not be limited to *NF1* mutant tumors, but may operate in other genetic contexts to promote hyperproliferation.

As a negative regulator of Ras signaling, Grb10 also modulates PI3K and MAPK signaling pathways, although our experiments involving untransformed MEFs and tumor cells suggest that the precise downstream effects may differ depending on context. *Grb10* knockdown in *Nf1* WT and null MEFs increased both ERK and Akt phosphorylation, consistent with Ras activation and somewhat symmetric activation of downstream PI3K and MAPK signaling arms of the Ras pathway cascade. This activation pattern contrasts with the signaling consequences of Grb10 restoration observed in tumor cells. In the tumor cell lines tested here, Grb10-mediated growth suppression is largely mediated by dampening MAPK rather than PI3K signaling, possibly reflecting intrinsic dependencies of these tumors on activated MAPK signaling. The Grb10 protein is phosphorylated by mTORC1, which stabilizes it and supports its function as a negative regulator of PI3K, thus completing a negative feedback loop that has been described in MEFs [21]. Consistent with this model, we found that *Grb10* silencing increased phosphorylated Akt levels, however phosphorylated ERK and Ras-GTP levels also increased, consistent with Ras activation. In *Nf1* null tumors, restoration of Grb10 reduced levels of ERK and Akt, although the magnitude of this effect was unequal between these pathways.

Grb10 possesses multiple functional domains and phosphorylation sites [21], however the functional roles of these areas are not well understood. Our data suggest that alterations in Grb10 phosphorylation sites influence the dynamics of the signaling, which are evident in the signaling differences observed between wildtype Grb10 and mutant Grb10 AA. While 989 cells expressing wildtype Grb10 demonstrate the predicted response to insulin stimulation characterized by transient increase in pERK levels, peaking at 5 minutes post-stimulation, and followed by attenuation of the signal by 15 minutes, 989 cells expressing the mutant Grb10 AA have a decreased phosphorylated ERK with serum starvation and more sustained ERK phosphorylation in response to stimulation (Fig 3D). Wildtype Grb10 normalizes the signal upon stimulation with insulin, while Grb10 AA decreases ERK phosphorylation after serum starvation, without affecting the dynamics of the signal. Furthermore, expression of the mutant Grb10 AA was associated with elevated Ras-GTP levels at 5 minutes, which is not concordant with ERK phosphorylation. This difference may reflect specific functions of the Serine 501/503 residues that are mutated in the Grb10 AA protein. Functional analysis of Grb10’s domains will enable better characterization of Grb10’s effects on signaling by Ras and its effectors, which may be context-dependent.

Given Grb10’s functional connection to well-described tumor promoting signal transduction pathways, the literature suggests that Grb10 function could contribute to cancer development. Grb10 has not previously been shown to be intrinsically oncogenic either *in vivo* or *in vitro*, nor have tumor-promoting functions of mTOR, the best understood phosphorylator of Grb10, been shown to be Grb10-dependent. However, mTOR clearly links proliferative signaling to protein translation and plays an important role in many cancers [43–46]. mTORC1-driven inhibition of the PI3K pathway signaling occurs through Grb10 [22], and this negative feedback mechanism from mTOR to PI3K signaling suggests a potential role for *Grb10* loss to promote cell proliferation. Although speculative, Grb10 likely functions in a complex network with mTOR and its other effectors, and its contribution to oncogenic signaling may depend upon both the signaling and cellular backgrounds.

Our data indicates that *Grb10*-associated tumorigenesis is conditional on *Grb10* loss cooperating with other tumor-promoting genetic events, as *Grb10* silencing alone failed to confer anchorage independent growth to MEFs. This is also consistent with the *Grb10* mutant phenotype *in vivo*, as *Grb10* knock-out mice do not spontaneously develop tumors [3]. Genetic loss was responsible for loss of *Grb10* expression in the majority of our tumors. In humans, the genetic basis for *GRB10* and *NF1* co-loss differs from the genetics in murine tumors, because the *GRB10* and *NF1* genes reside on different chromosomes (chromosome 7 and 17, respectively). Therefore, loss of these genes in human cancers will be independent events rather than occurring by a single genetic event as suggested by our mouse model. In addition, non-genetic alternative mechanisms might be operational in human cancers promoted by *GRB10* and *NF1* co-loss. Indeed, our data suggest the presence of post-translational mechanisms mediating *Grb10* loss, as among our tumor cell lines one tumor (the 867 tumor cell line) demonstrated *Grb10* transcript levels comparable to normal control tissues but lacked detectable Grb10 protein. However, there are likely post-translational mechanisms mediating *Grb10* loss, as among our tumor cell lines one tumor (the 867 tumor cell line) demonstrated *Grb10* transcript levels comparable to normal control tissues but lacked detectable Grb10 protein. Given multiple potential mechanisms for Grb10 protein function, Grb10 protein levels may be more informative than genetic or transcript analysis alone in determining *GRB10* status in human cancers.

This work illustrates that *Nf1*-mediated tumorigenesis can be promoted by loss of another negative regulator of Ras signaling, and raises the possibility that other negative regulators may contribute to *Nf1*-driven tumorigenesis in other contexts. Potential candidates for these alternative negative regulators might include some Grb protein family members. This family includes Grb7, Grb10 and Grb14, which are structurally related multi-domain adapter proteins with overlapping and distinct functions. All three family members share a polyproline stretch, PH, RA, SH2 and PBS domains, however their functions differ. While Grb7 was shown to play a role in adhesion/migration by associating with membrane regions of focal adhesion kinases and the EphB1 receptor [47], Grb10 and Grb14 have been implicated in the regulation of insulin receptor and insulin growth factor receptor signaling, and potentially other receptor tyrosine kinases [48]. Mouse models of Grb10 and Grb14 demonstrate the overlapping, distinct, and tissue-specific *in vivo* functions of Grb10 and Grb14 in insulin signaling regulation. Specifically, Grb14 has IR-mediated growth inhibitory effects in the liver and retina, whereas Grb10 plays a role in insulin signaling regulation in the muscle and adipose tissue [48–50]. As such, *Grb10* mutant mice with loss of the expressed allele have a 30% increase in body size, whereas *Grb14* ^*-/-*^ mice are of normal size. Recent structural studies have also shown that Grb10 and Grb14 have different affinities for binding RTKs, with Grb10 having a higher affinity of binding to phosphor-inositol phosphates on the membrane through its PH domain, while Grb14 has greater affinity for binding Ras molecules [18,51]. In our studies, Grb10 was found to be uniformly lost in the mouse tumors but we found no evidence of a compensatory increase in the levels of other Grbs, including Grb14, the most closely related Grb family member. Perhaps this is not surprising, given that most of the tumors from the irradiated *Nf1* ^*+/-*^ mice are sarcomas derived from muscle tissue, where Grb10 has a more prominent role than Grb14 [27,52].

This work describes a previously unknown role for an imprinted gene as a tumor suppressor. Conceptually, restricting gene expression to originate from a single allele creates haploinsufficiency, which can expose an organism to disorders caused by loss of protein expression, cancer being an example. Presumably imprinting confers a compelling organismal advantage to offset this risk, and recent data support the idea that *Grb10* imprinting is evolutionarily driven by nutrient utilization during development, and *Grb10* influences proportions of lean and fat tissues during development [53]. An intriguing connection between *Grb10* and tumorigenesis suggests that genetic mechanisms that initially developed to confer an advantage in energy storage and utilization during development might in later life limit a cell’s ability to suppress inappropriate proliferation.

Imprinting means that loss of either allele will impact different cell types variably, and that the cellular context determines the consequence of genetic loss. This feature of imprinted genes has implications for the types of tumors that can be expected to arise after *Grb10* loss. The breeding schema of our mouse model produced F1 mice inheriting the mutant *Nf1* copy paternally, and subsequent co-loss of *Grb10* in *cis* with *Nf1* from the maternal copy of chromosome 11 is equivalent to *Grb10* nullizygosity in non-CNS tissues, where only the maternally-inherited *Grb10* allele is expressed. This pattern of loss does not produce *Grb10* nullizygosity in tissues expressing the paternal *Grb10*, for example tissues of the nervous system. In our breeding schema, the paternal *Grb10* allele is located *in cis* with the mutant *Nf1* allele, and we postulate that the *Grb10* imprinting unique to tissues of the nervous system confers resistance to tumorigenesis in the nervous system. Indeed, irradiated F1 mice in our mouse models do not develop CNS tumors [14], despite the fact that optic pathway gliomas (OPGs) are a type of CNS tumor that arises in 15–20% of children with NF1 [54]. The genetic factors responsible for OPG development in NF1 are not fully understood, however our experimental model provides a robust context in which to assess possible tumor promotion by loss of the imprinted paternal *Grb10* allele. In prior studies, *Grb10* has been proposed as a candidate modifier gene in *Nf1* mutant mice [32,35]. Analysis of tumors from our mouse models supports this role of *Grb10* and sheds light on the mechanism and consequences of its loss. Additional experiments in *Grb10* mutant mice will also be important for further elucidating the function of this protein *in vivo*. Apart from the development of malignant tumors in the *Nf1* mutant mouse background, genetic modifiers may hold broader relevance for the *NF1* mutant phenotype in humans. The severity of Neurofibromatosis I varies amongst family members sharing a germline mutation [55], and our data supports the concept that imprinted alleles function as disease modifiers and may be involved in mediating this variability. Candidate genetic modifiers have been identified in multiple sporadic human cancers [56,57]. Interestingly, polymorphism analysis of gliomas in NF1 patients found correlation between specific polymorphisms in the human adenylate cyclase 8 gene with glioma development in a sex-specific manner [58]. Sex-specific modifiers are driven by the sex of the affected individual and fundamentally differ from imprinted modifiers, which are defined by the parental allele involved. Finally, imprinted genes may influence an individual’s susceptibility to tumor development as well as potentially other diseases and represent a novel genetic mechanism that defines both normal physiology and disease.

## Materials and Methods

### Ethics statement

Tumor samples obtained from previously described mouse models were analyzed. All animal procedures were approved by the UCSF IACUC (Approved protocol numbers: AN078941 and AN080665). These practices conform to regulations defined by the Animal Welfare Act and the US Department of Agriculture.

### Cell lines and culture conditions

Mouse tumor cell lines were grown as previously described [40]. Cell lines used were established from tumors arising in irradiated *Nf1* ^*+/-*^ F1 mice [14,15,59]. Human cell lines were obtained from ATCC.

Retroviral vectors including HA-tagged retroviral WT-Grb10 and Grb10-AA (S501A–S503A) constructs were kindly provided by Dr. Yonghao Yu, (University of Texas Southwestern Medical Center). To generate retrovirus Ecopack packaging (5 μg) plasmids were co-transfected with the Grb10 plasmids (10–15 μg) into HEK 293 T cells using lipofectamine 2000 (Invitrogen). Lentivirus vectors including Flag-tagged WT-Grb10 and MEKDD constructs were cloned as described in the section Gateway Cloning.

To generate lentivirus, Grb10, MEKDD or GFP (9μg) plasmids were co-transfected with packaging plasmids Δ8.9 (9 μg) and VSVG (4.5 μg) into HEK293T cells using lipofectamine 2000 (Invitrogen).

Two days after transfection, retroviral or lentiviral supernatants were harvested and filtered. Recipient cells were infected in the presence of antibiotic-free, serum-containing medium supplemented with 8 μg/ml polybrene. Following infection, cell lines stably expressing the Grb10 constructs were selected using puromycin. Stable expression was confirmed by Western analysis to visualize the HA tag.

### Cell counting

Cells from all experimental groups were plated in triplicates at the same density (ranging from 50,000 to 150,000 cells/well in 6-well plates depending on the experiment). Cells were trypsinized and re-suspended in 2 mLs of complete media. Cell counting and assessment of viability by trypan blue staining were performed using an automated cell counter, Vi-CELL XR (Beckman Coulter, Fullerton, CA).

### Anchorage independent growth assay

Cell were plated 4 x 10^4^ cells per 6 cm plate in 1x DMEM as previously described [40]. One day following plating, the plates were treated with drug to indicated concentration, or DMSO carrier as control, diluted in DMEM.

### Western blotting

For signaling pathway Western blots, cells were collected at 70% confluence. Cells were washed twice in PBS and lysed in RIPA lysis buffer (1% Sodium Deoxycholate, 0.1% SDS, 25 mM Tris, 150 μM NaCl, 1% Triton-X, 0.2 mM EDTA, 10 mM NaF, 1 mM Sodium Vanadate, 10 nM Calyculin A, Protease Inhibitors). Lysate protein concentration was determined by Pierce BCA Protein Assay Kit (Thermo Scientific). 20–50 μg of cell lysate was run on SDS-PAGE 10–20% gradient gels (Novex). Most antibodies were purchased from Cell Signaling Technologies. Primary antibodies were diluted at 1:2000, and included Beta-actin (Cat No. 4967L), phospho-S473-Akt (Cat No. 4060), phospho-S6 (Cat No. 2211), phospho-p44/42 MAPK (Cat No. 4376), IRβ (Cat No.3025), Flag (Cat No. 8146), Phospho-Tyrosine (Cat No. 8954), HA (Cat No.2367). Anti-Grb10 and EGFR antibodies were purchased from Santa Cruz (Cat No. sc-1026, and sc-31157 respectively). Anti-IGFR antibody was purchased from Millipore (Cat No. 05–656). Secondary antibodies were diluted at 1:2000 and included anti-rabbit IgG-HRP (Cat No. 7074) and anti-mouse IgG-HRP (Cat No. 7076). NIH Image 1.49j or Li-Cor Image Studio Lite 4.0.21 softwares were used for densitometric quantification of phosphorylated proteins and receptor levels. For the quantification of receptor levels in the stimulation experiments, receptor band intensities were normalized to Actin as an internal reference. Subsequently all the values were normalized to the initial experimental condition: Luc/ time point “0”. For quantification of phosphorylated protein levels, Total AKT, pAKT, Total ERK, pERK intensity levels were first all normalized to Actin as an internal control, and the normalized values were used to calculate the ratio of pAKT/Total AKT and pERK/Total ERK. Quantifications displayed under the corresponding bands correspond to the values obtained for the represented experiment.

### PCR array

RNA was extracted with the RNAeasy kit (Qiagen). RNA purity was assessed by UV spectrophotometry using a Nanodrop p1000 (Thermo Scientific). cDNA was generated from 1 μg RNA with the Qiagen RT2 First Strand Kit. Genomic DNA was removed with the Qiagen Genomic DNA Elimination Mix. Reverse-transcription mix was added to purified RNA, mixed with the RT2 SYBR Green Mastermix and added to the 96-well PI3K (Cat No. PIMM058A) PCR array plates per Qiagen protocol. RT-PCR was performed over 40 cycles on a Stratgene MX3000P qPCR system. CT values were exported into a Microsoft Excel spreadsheet and uploaded into the Qiagen RT2 Profiler PCR Array Data Analysis Webportal software (v3.5) for analysis (http://pcrdataanalysis.sabiosciences.com/pcr/arrayanalysis.php). Data QC was verified and housekeeping genes were selected for data normalization. Fold change was calculated and used to generate the heat map.

### BrdU incorporation analysis

Equal numbers (250,000 cells) of WT and *Nf1* null MEFs expressing shRNA against Luciferase (Luc) or Grb10 were plated in 6-well plates. BrdU was added to the culture medium overnight, and the cells were trypsinized and harvested the next day. The cells were fixed and stained with APC-labeled anti-BrdU antibodies as recommended by the BD BrdU flow kit protocol (BDB552598). Percent BrdU incorporation was assessed by flow cytometric analysis (FACSDiva LSRII). For mitotic enrichment 250,000 cells were plated in 6-wells plates and treated with 25 μM MG132 for 2 hrs prior to labeling with BrdU as discussed previously.

### Immunoprecipitation

Cells were lysed with modified Ripa buffer (50 mM Tris-Cl, pH 7.5, 150 mM NaCl, 1% Nonidet P40, 0.5% sodium deoxycholate, 0.1% SDS, 10 mM NaF, 1 mM Sodium Vanadate, 10 nM Calyculin A, Protease Inhibitors). Lysates were centrifuged at 15,000 g for 20 min at 4°C, and protein concentration was determined by Pierce BCA Protein Assay Kit (Thermo Scientific). 100μg of protein was diluted in a total of 200μl of modified Ripa buffer then precleared with protein A agarose beads (Life Technologies) at 4°C for 1 hr. The supernatants were then incubated with 1 μg of anti-insulin receptor antibody (Cell Signaling) at 4°C overnight. Twenty microliters of protein A agarose beads were then added/ml of lysate, and incubated at 4°C for 1 hr. The agarose beads (with the antibody-protein complex) were then collected by centrifugation (10 min, 14,000 g, 4°C). Supernatants were discarded and beads were washed 3 × with PBS. Finally, the beads were re-suspended in 20 μl of 2 × sample buffer, boiled for 5 min, electrophoresed on 10% Tris-glycine gels (Novex).

### Statistical analysis

Prizm v.4 (GraphPad) was used to calculate paired student’s t tests. Experiments were performed at least three times and means with p <0.05 were considered statistically significant.

See S1 Text for additional experimental procedures.

## Supporting Information

S1 Text Supplementary materials and methods. (DOCX)

S1 Fig *Grb7* and *Grb14* levels are reduced in *Nf1* mutant tumor cell lines whereas *Grb2* levels are variable. QPCR analysis of *Grb14* (A) *Grb7* (B) and *Grb2* (C) mRNA normalized to β-Actin reveals that *Grb7* and *Grb14* (closely related to *Grb10*) are also reduced in *Nf1* null tumor lines compared to adult normal tissues, whereas *Grb2* levels are comparable to the adult controls.
(TIF)

S2 Fig *Grb10* knockdown increases proliferation as assessed by BrdU labeling. A. WT and *Nf1* null MEFs expressing shRNA against Grb10 exhibit increased BrdU labeling compared to MEFs with Luciferase knockdown. Scatter plot shows normalized data to the average luciferase control. Data from 3 independent experiments shown, (t-test, **p<0.001, and *p<0.05). B. WT and *Nf1* null MEFs expressing shRNA against Luciferase (control) or Grb10 were treated with MG132 (25 μM for 2 hours prior to BrdU labeling) to enrich for mitotic cells. Graph shows percent BrdU positive relative to total in WT and *Nf1* null MEFs with shRNA against luciferase or Grb10 (t-test, ***p<0.0001). C. WT and *Nf1* null MEFs expressing shRNA against Luciferase (control) or Grb10 were cultured as in (B) and treated with MG132 (25 μM for 2 hours) then washed and left in normal growth medium then counted after 48 hrs. Graph shows total number of cells after 48 hours (t-test, *<0.05, and ***p<0.0001).
(TIF)

S3 Fig *Grb10* silencing in MEFs increases insulin receptor phosphorylation. A. WT and *Nf1* ^*-/-*^ MEFs expressing shRNA against *Trp53* and shRNA against *Luciferase* (Luc control) or *Grb10* were serum starved overnight, then stimulated with insulin. Whole cell lysates were collected at shown timepoints (minutes after insulin addition) and immunoprecipitation with an anti-insulin receptor antibody was performed and samples were analyzed for phospho-tyrosine levels. Immunoblotting on total lysates with anti-insulin receptor antibody was performed as a control.
(TIF)
