# Correction: Monoallelic Loss of the Imprinted Gene *Grb10* Promotes Tumor Formation in Irradiated *Nf1+/-* Mice

**DOI:** 10.1371/journal.pgen.1005327

**Published:** 2015-06-15

**Authors:** Rana Mroue, Brian Huang, Steve Braunstein, Ari J. Firestone, Jean L. Nakamura

The corresponding author for this article is not listed. The corresponding author is Jean L. Nakamura (Jean.Nakamura@ucsf.edu).

The editor’s affiliation is missing. Bruce Korf’s affiliation is: University of Alabama, Birmingham School of Medicine, United States of America.
