# Supplementary material for: Monoallelic Loss of the Imprinted Gene Grb10 Promotes Tumor Formation in Irradiated Nf1+/- Mice
Source: PLoS Genet. 2015 May 22;11(5):e1005235. doi: 10.1371/journal.pgen.1005235 (PMC4441450; doi:10.1371/journal.pgen.1005235)
Supplement: S1 Text — (DOCX) [file pgen.1005235.s001.docx]

**SUPPLEMENTARY MATERIALS AND METHODS**

RT-PCR

Total RNA was isolated with QIAGEN RNeasy® Mini Kit. For cDNA synthesis, 50 ng of total cellular RNA was used to synthesize cDNA using SuperScript III First-Strand Synthesis System (Invitrogen). Two sets of primers for Grb10, Grb2, Grb7 and Grb14 and a previously validated set of β-actin primers were used in the reactions.

| Grb14 | CCGGGACCTGAAAATGCTCT | ATTGCAGGCGCTTCTACCTT |
| --- | --- | --- |
| Grb14 (2) | GCTTTCGGTTGCTGTTGAGG | CCGATGGAGAGCCATGTTCA |
| Grb7 | CGGTGCATTGTGAGGAGTCT | GGTAGAAAGGCTATCCCGCC |
| Grb7 (2) | TCTGAGCCCGACTCATCTCA | GAGTCTCAGGAGGGGTAGCA |
| Grb2 | AACATCCGTGTCCAGGAACC | AAGTCTCCTCTGCGAAAGCC |
| Grb2 (2) | CTCTCTTGGCAGTGGGTTGT | CAGGCACGTGACTACAGAGG |
| Grb10 | GTGGTGGAGATTCTAACCGACA | ACCTCTCTAATCCCAGTTGTGG |
| Grb10(2) | ACAGGATCATCAAGCAACAA | TCTTTGTGAAGTCCAATAAC |
| b-actin | TTCGTTGCCGGTCCACACCC | TTTGCACATGCCGGAGCCGT |

#### All primers were made by Integrated DNA Technologies (IDT®). Real-time PCR was performed using Applied Biosystems 7900HT Fast Real-Time PCR System machine, according to the manufacturer's instructions. QuantiFast SYBR Green Kit (Qiagen) was used for the PCR reaction. PCR data analysis was performed with SDS v2.3 Software (Applied Biosystems, Life Technologies). Statistical analysis of the data was done in GraphPad Prism 5 and plotted as mean levels of *Grb* transcripts (4 replicates) normalized to β-Actin. The experiment was repeated 3 times and representative values are shown in figures.

**Hairpin design and MEF transduction:**

Lentiviral shRNA expression vector pCDH LMN-mCherry was created by excising the miR30-PGK-NeoR-IRES-mCherry cassette from LMN-mCherry (REF) Bgl II(blunt)-Sal. The resulting fragment was then ligated into into pCDH-MCS-T2A-copGFP-MSCV (Systems Biosciences, #523A) prepared by the sequential process of digestion with Eco RI, blunting with Klenow, and digestion with Sal I. Hairpin ultramers were designed as previously described [47] using the specified selection criteria for optimal efficiency. The 4nmol ultramers were made by IDT (Integrated DNA Technologies Inc.) and re-suspended to a final concentration of 10µM. The following shRNA ultramers are listed in their order of knockdown efficiency with 55 and 564 chosen as the best hairpins:

| **mmGrb10-hp-55** | TGCTGTTGACAGTGAgcgAcagtctgatactgatactgcaTAGTGAAGCCACAGATGTAtgcagtatcagtatcagactgcTGCCTACTGCCTCGGA |
| --- | --- |
| **mmGrb10-hp-564** | TGCTGTTGACAGTGAgcgCgagcaaattcttattcagaaaTAGTGAAGCCACAGATGTAtttctgaataagaatttgctctTGCCTACTGCCTCGGA |
| **mmGrb10-hp-579** | TGCTGTTGACAGTGAgcgCcagaaagaattatgcgaagtaTAGTGAAGCCACAGATGTAtacttcgcataattctttctgaTGCCTACTGCCTCGGA |
| **mmGrb10-hp-945** | TGCTGTTGACAGTGAgcgCcaaagcgaagaccgagatgaaTAGTGAAGCCACAGATGTAttcatctcggtcttcgctttgtTGCCTACTGCCTCGGA |
| **Trp53** | [47] |

Ultramers were further diluted and pooled to create a final concentration 10nM of each hairpin (up to 10 in the pool). 5’ XhoI and 3’ ECORI digestion sites flanking the ultramers were PCR-added using the following primers:

| **miR30 forward** | **miR30 reverse** |
| --- | --- |
| 5’CAGAAGGCTCGAGAAGGTATATTGCTGTTGACAGTGAGCG 3’ | 5’CTAAAGTAGCCCCTTGAATTCCGAGGCAGTAGGCA 3’ |

PCR reaction was performed using the GC-rich kit (Invitrogen, Life Technologies Cat No.12337-016), followed by digestion with EcoRI and XhoI at 37 C for 3 hours. The product was then column purified using the Qiagen kit (Qiagen© Cat No. 28104), and the purified constructs were cloned into into pCDH MCS T2A copGFP vector (which includes a neo-selectable cassette). This vector was digested similarly to the PCR product at 37C for 3 hours. The digested vector was gel purified on a 0.8% agarose gel and CIP-treated, and both the digested vector and shRNA insert were ligated overnight using T4 ligase (New England BioLabs, Cat No. M0202). DH5 alpha competent cells (Invitrogen, Life Technologies, Cat No. 18265-017) were transformed with 5µl of the ligation reaction and plated on to Ampicillin 100ug/ml plates (TEKnova, Cat No. L1004). After overnight incubation at 37C, colonies were picked, amplified overnight by shaking in LB broth, and shRNA plasmids were isolated using Qiagen MiniPrep Kit (Qiagen©, Cat No. 27104) Clones were sequenced and verified using the following primer:

5’-CCCTTGAACCTCCTCGTTCGACC-3. After positive clones were identified and expanded, they were used to transfect HEK 293T cells using PSPAX and VSVG as packaging plasmids (PSPAX-10 µg, VSVG - 2µg, LMN shRNA vector – 10 µg) in 10mL pen-strep free media. Viral medium was filtered and placed on top of the target cells two days post-transfection and resupplied daily until fluorescent cells appeared. Selection using 1.0 mg/mL working concentration of G418 was started typically a day after observing fluorescence.

**Gateway Cloning**

Lentivirus Destination vectors modified from Invitrogen’s Gateway system (pLenti-CMV-Puro-DEST) were kindly provided by Frank McCormick (UCSF); pDONR was available commercially (Invitrogen). For generation of WT-Grb10 expression construct, pRK5-Flag-Grb10 (kindly provided by David Sabatini, Massachusetts Institute of Technology, Cambridge, MA; Addgene plasmid 37481) was used as a PCR template, and for MEKDD expression construct, pDONR223-MEKDD (kindly provided by Benjamin Braun, UCSF; Addgene plasmid 31202) was used as a template, and the respective PCR amplicons were recombined into pDONR using BP Clonase (Invitrogen) to generate Entry vectors.

|  | **FW** | **Rv** |
| --- | --- | --- |
| **Grb10** | GGGG**-ACA-AGT-TTG-TAC-AAA-AAA-GCA-GGC-TTC**-ACC-ATG-gat-tac-aag-gat-gac-gac-gat-aag-ATG-GCT-TTA-GCC-GGC-TGC-CCA | GGGG-ACC**-ACT-TTG-TAC-AAG-AAA-GCT-GGG-TCC**-TCA-TAA-GGC-CAC-TCG-GAT-GCA |
| **MEKDD** | GGGG**-ACA-AGT-TTG-TAC-AAA-AAA-GCA-GGC-TTC**-ACC-ATG-GAT-TAC-AAG-GAT-GAC-GAC-GAT-AAG-ATG-CCC-AAG-AAG-AAG-CCG-ACG | GGGG-ACC**-ACT-TTG-TAC-AAG-AAA-GCT-GGG-TCC**-TTA-GAC-GCC-AGC-AGC-ATG-GGT |

PENTR-Grb10 and PENTR-MEKDD constructs were recombined into pLenti-CMV-Puro-

DEST using LR Clonase (Invitrogen). Recombination products were transformed in DH5 alpha competent cells and plated on Amp plates. Colonies were picked and sequenced and verified before amplification and use to generate lentiviral particles.

**Cbioportal queries and data mining**

The data portal, cBioPortal for Cancer Genomics [41, 42] was interrogated using the available R software package (Version i3863.0.2) to investigate the incidence of *GRB10* and *NF1* mutations in the 56 studies of human cancers available on cBioPortal.

**Illumina SNP Array and *Grb10* Microsatellite analysis**

Genomic DNA was isolated as previously described [13]. Genomic DNA isolated from control tail and radiation-induced tumors were hybridized to the Illumina Mouse Medium Density Linkage Panel per the manufacturer’s instructions. The SNP genotype outputs of radiation-induced solid tumors were compared between tumor and normal control, and LOH events at each SNP locus were defined as those events where a SNP that was heterozygous in a normal (F1) control became homozygous in the tumor sample.

Loss of heterozygosity at the *Grb10* locus was assessed by analysis of PCR-amplified fragments across a microsatellite region extending between 11927436-11927475 with 20XAC repeats, identified using the Ensembl Genome Browser.  The primers used for the amplification of the microsatellite locus were as follows:

| FW: FAM-tag+ctcaaatctgtatagtctcaggtctca | RV: GTGTCTTgcctcccatttccttattcc |
| --- | --- |

Fragment analysis was performed by the UCSF Genomics Core Facility [13]. B6, 129 and F1(B6x129) tail DNA was used as controls.
