# Supplementary figures and images for: Monoallelic Loss of the Imprinted Gene Grb10 Promotes Tumor Formation in Irradiated Nf1+/- Mice
Source: PLoS Genet. 2015 May 22;11(5):e1005235. doi: 10.1371/journal.pgen.1005235 (PMC4441450; doi:10.1371/journal.pgen.1005235)

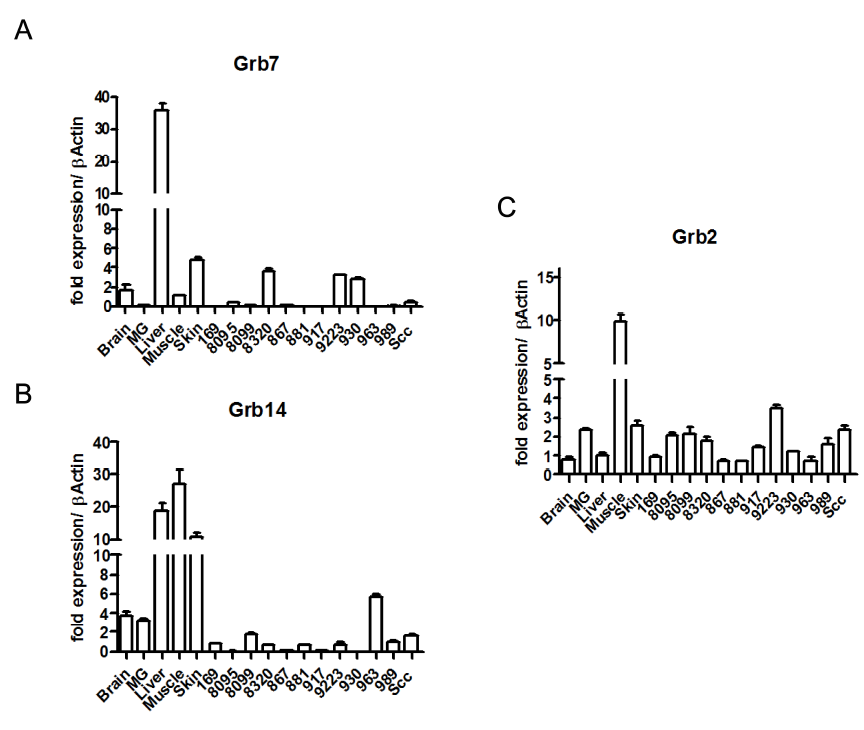

Supplement: S1 Fig — QPCR analysis of Grb14 (A) Grb7 (B) and Grb2 (C) mRNA normalized to β-Actin reveals that Grb7 and Grb14 (closely related to Grb10) are also reduced in Nf1 null tumor lines compared to adult normal tissues, whereas Grb2 levels are comparable to the adult controls. (TIF) [file pgen.1005235.s002.tif]

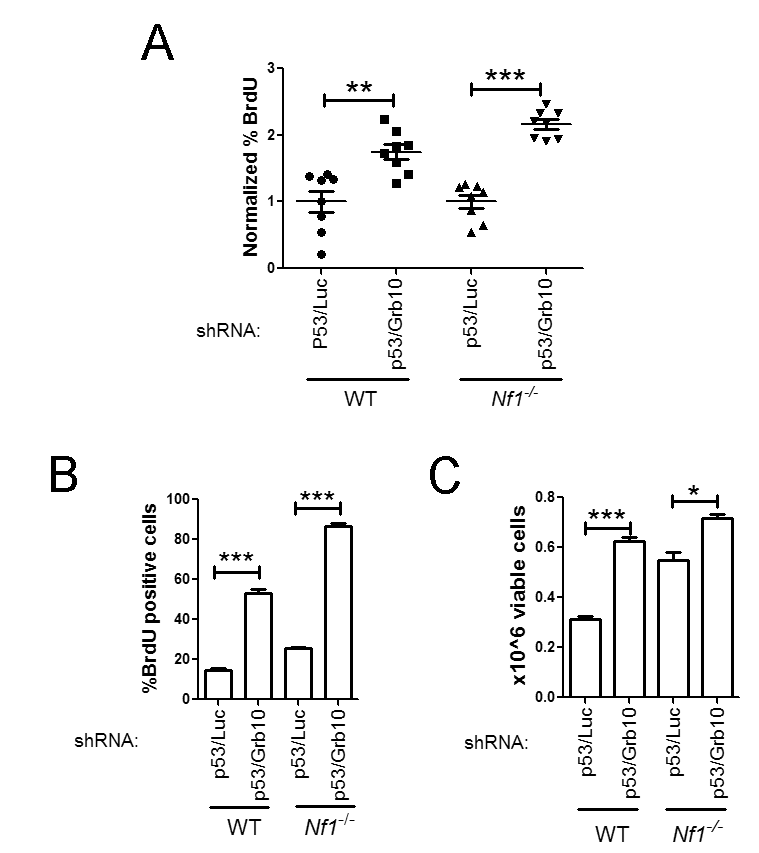

Supplement: S2 Fig — A. WT and Nf1 null MEFs expressing shRNA against Grb10 exhibit increased BrdU labeling compared to MEFs with Luciferase knockdown. Scatter plot shows normalized data to the average luciferase control. Data from 3 independent experiments shown, (t-test, **p<0.001, and *p<0.05). B. WT and Nf1 null MEFs expressing shRNA against Luciferase (control) or Grb10 were treated with MG132 (25 μM for 2 hours prior to BrdU labeling) to enrich for mitotic cells. Graph shows percent BrdU positive relative to total in WT and Nf1 null MEFs with shRNA against luciferase or Grb10 (t-test, ***p<0.0001). C. WT and Nf1 null MEFs expressing shRNA against Luciferase (control) or Grb10 were cultured as in (B) and treated with MG132 (25 μM for 2 hours) then washed and left in normal growth medium then counted after 48 hrs. Graph shows total number of cells after 48 hours (t-test, *<0.05, and ***p<0.0001). (TIF) [file pgen.1005235.s003.tif]

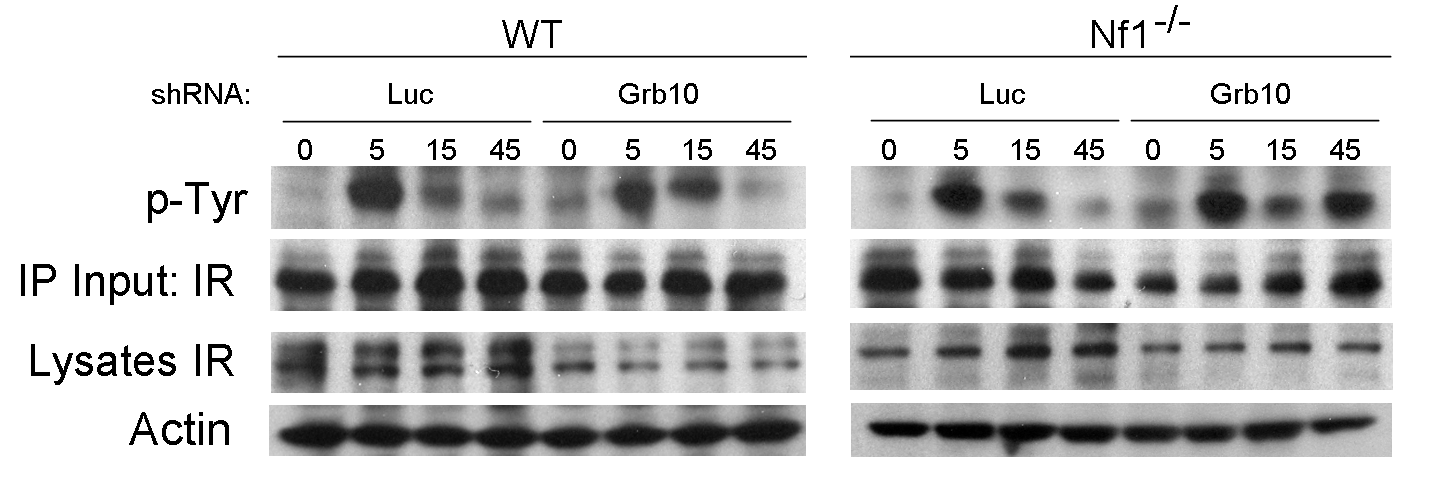

Supplement: S3 Fig — A. WT and Nf1 -/- MEFs expressing shRNA against Trp53 and shRNA against Luciferase (Luc control) or Grb10 were serum starved overnight, then stimulated with insulin. Whole cell lysates were collected at shown timepoints (minutes after insulin addition) and immunoprecipitation with an anti-insulin receptor antibody was performed and samples were analyzed for phospho-tyrosine levels. Immunoblotting on total lysates with anti-insulin receptor antibody was performed as a control. (TIF) [file pgen.1005235.s004.tif]
